# Effectiveness of tailored COVID-19 messages for vulnerable Australians: A study protocol

**DOI:** 10.1371/journal.pone.0280865

**Published:** 2023-01-27

**Authors:** Megan Jepson, Nathan Williams, Terry P. Haines, on behalf of the PIVC (Pandemic Information for Vulnerable Communities) Group

**Affiliations:** 1 School of Primary and Allied Health Care, Monash University Peninsula Campus, Melbourne, Victoria, Australia; 2 Department of Paramedicine, Monash University Peninsula Campus, Melbourne, Victoria, Australia; All India Institute of Medical Sciences - Rishikesh, INDIA

## Abstract

Multiple approaches can be used to communicate public health messages through mass media. It is unclear which approaches are superior for meeting the needs of the general community along with vulnerable population subgroups. To compare different public health strategy communication approaches for influencing the COVID-safe behavioural intentions of both community and vulnerable population subgroups. This study will conduct three concurrent ‘helix’ randomised controlled trials with Latin square sequencing and factorial intervention allocation to assess the effectiveness of different communication strategies amongst the Australian general community and six subgroups that are considered vulnerable to contracting, transmitting or experiencing severe consequences of COVID-19 infection. Communication approaches being compared include: the format of communication (written versus video), who is providing information (general practitioner, politician, community-representative), what is said and how it is delivered (direct information provision versus conversational approach) and the visual content of video messaging (animation versus ‘talking head’). Recruited participants will be randomly allocated to receive a specific combination of health messaging strategies using six different COVID-19 context areas. Outcomes will be assessed in a survey using behaviour intention questions, and questions surrounding level of agreement with feeling represented in the health messaging strategy. These trials will use a unique research approach to provide an experimental evidence base to help guide development of impactful and inclusive COVID-19 and related public health messaging. All three trials are registered with the Australian New Zealand Clinical Trials Registry (ANZCTR). Trial 1: Update and impact of Government recommendations about COVID-19 (coronavirus)-Stage 3, Trial 1, vulnerable subgroup populations (ACTRN12622000606785). Trial 2: Update and impact of Government recommendations about COVID-19 (coronavirus)-Stage 3, Trial 2, community group (ACTRN12622000605796). Trial 3: Update and impact of Government recommendations about COVID-19 (coronavirus)-Stage 3, Trial 3, What communication strategy is most effective for both vulnerable and community group populations? (ACTRN12622000617763).

## Introduction

### Background and rationale

Informative public health messages have been widely used as a part of the global COVID-19 response [1, 2]. Research conducted since the beginning of the COVID-19 pandemic has highlighted the disparity that exists in health outcomes of the general population compared with vulnerable communities [3–8]. It has been argued that public health messages need to address the various needs (including healthcare, communication and cognitive considerations), as well as the health literacy and sociocultural factors relevant to vulnerable communities, in order to reach these communities effectively [7, 9–12]. There are several dimensions by which public health messaging can be varied, which may have an impact on its effectiveness. These can include: the format of communication (written versus video), who is providing information (general practitioner, politician, community-representative), what is said and how it is delivered (direct information provision versus conversational approach) and the visual content of video messaging (animation versus ‘talking head’).

How people access information has changed rapidly in recent years. It is estimated that by the end of 2022, internet video traffic will be 82% of all consumer internet traffic (up from 73% in 2017) [13]. This highlights the potential instinctive preference for consumers to choose to receive health information via video format compared with written information. Previous research has indicated that older adults respond more positively to information presented in video format compared to equivalent written information and tend to prefer video format via television news programs [14, 15]. Similarly, younger people who are more exposed to social media platforms, such as YouTube and Facebook, will be more likely to view public health messaging in video format, as these are largely video based platforms which allow users to quickly and easily view and share content [14, 16]. While research has aimed to identify the preferred format of receiving health information (video versus written), the effectiveness of either has yet to be strongly established, and in Australia, the Commonwealth Department of Health has continued to use both methods during the pandemic [17]. Further research is required to better establish not only the preference, but more importantly the effectiveness of written public health messaging compared to video based public health messaging, for the general Australian community.

The issue of ‘who’ provides the public health message is an important consideration in ensuring that it is effective [18, 19]. During the COVID-19 pandemic there has been a range of spokespeople providing public health messaging, including politicians, jurisdictional medical officers, scientists, general practitioners and other health professionals, and community representatives [1, 20]. Previous work has shown trust to be a key factor in the acceptance and adoption of COVID-safe behaviours such as physical distancing, staying at home when you are unwell and other prevention guidelines [19, 21]. These studies suggest people tend to have a higher level of trust in scientists and health professionals conveying COVID-19 health recommendations (compared to politicians), and are more likely to implement COVID-safe behaviours recommended to them by scientists, compared to recommendations provided by politicians who they trust less [19, 21–23]. Trust in health professionals, such as general practitioners, has also been seen to impact vaccine uptake in minority ethnic groups in the United Kingdom [24]. Similarly, some qualitative work has indicated that culturally and linguistically diverse groups respond more positively to health messages delivered by community representatives, as they are more trusted [25]. During the COVID-19 pandemic, the Australian Government created health information videos presented by various representatives within the community. Some of these included, government representatives (for example, chief medical officers), general practitioners and community representatives. Although ‘who’ presents the message has been varied, it is not yet known ‘who’ is preferred or how effective these videos are in eliciting positive behavioural change in both the general Australian community and vulnerable subgroup populations. There is also further need to understand how bringing about behaviour change for vulnerable subgroup populations may differ from the ways that this is achieved for the general Australian community.

How a given spokesperson communicates a public health message may also be of importance. Some videos may take a direct, information provision approach, such as a public service announcement [26]. Others might use a more conversational approach, such as providing relatable advice, or through the use of a narrative [27]. These approaches and many others have been used by advertisers, health authorities and governments internationally throughout the COVID-19 pandemic [28–30]. It has been suggested that direct information provision strategies may not achieve their desired effect, particularly in areas with low levels of trust in the government [31]. Conversely, communication that is empathetic and uses a narrative style is argued to be a more effective approach [32, 33]. While this may be true for the general community, research regarding which information provision approach has the greatest impact and effectiveness for vulnerable subgroup populations is still lacking.

A further dimension to be considered in public health messaging is the visual content or imagery used while the message is being conveyed. Some videos use a piece to camera, or ‘talking head’ approach, where a person of relevance presents information as if talking to the audience, generally remaining on-screen for the majority of the video [34]. An alternative approach is showing an animation as the visual content, with voice over. The Australian Government has used both methods during the COVID-19 pandemic [34, 35]. The talking head approach has been argued to have varied effectiveness, depending on who is delivering the message and the public’s level of trust in that person [31]. Previous research has indicated that using animation can lead to increased COVID-19 knowledge, compared with no intervention [36]. It has also been suggested that increased exposure to animation leads to increased recall of the health information [37]. Therefore, it would be useful to ascertain which visual content has the greatest impact on vulnerable populations.

Public health messaging campaigns have many dimensions to their success. Understanding these dimensions in greater detail is important in ensuring an effective and cost-effective campaign is produced, leading to the greatest uptake in positive health behaviours. This research will investigate the effects of delivering COVID-19 public health messaging to the Australian community as well as six vulnerable subgroup populations in multiple formats. Differences include written versus video-based information provision as well as differences in the production of each video. Videos will differ in who presents the information (general practitioner or a community group representative), how the message is conveyed (direct information provision or conversational approach), and what is seen on-screen (animation or talking head).

### Objective

This protocol outlines the design and methods for three concurrent ‘helix’ randomised controlled trials to investigate how to best deliver a health message to all Australians (vulnerable and general community groups). Specifically, we seek to investigate the importance of:

1. Who provides public health messages (general practitioner, politician, community leader)
2. The visual imagery used in public health messages (talking head versus animation)
3. The format of public health messages (written versus video)
4. How a public health message is delivered (conversation training versus information provision)

on the outcomes of intention to follow COVID-safe behaviours, likelihood of encouraging others to follow COVID-safe behaviours, and perception of being represented in the public health message.

## Materials & methods

### Design

**Fig 1:**
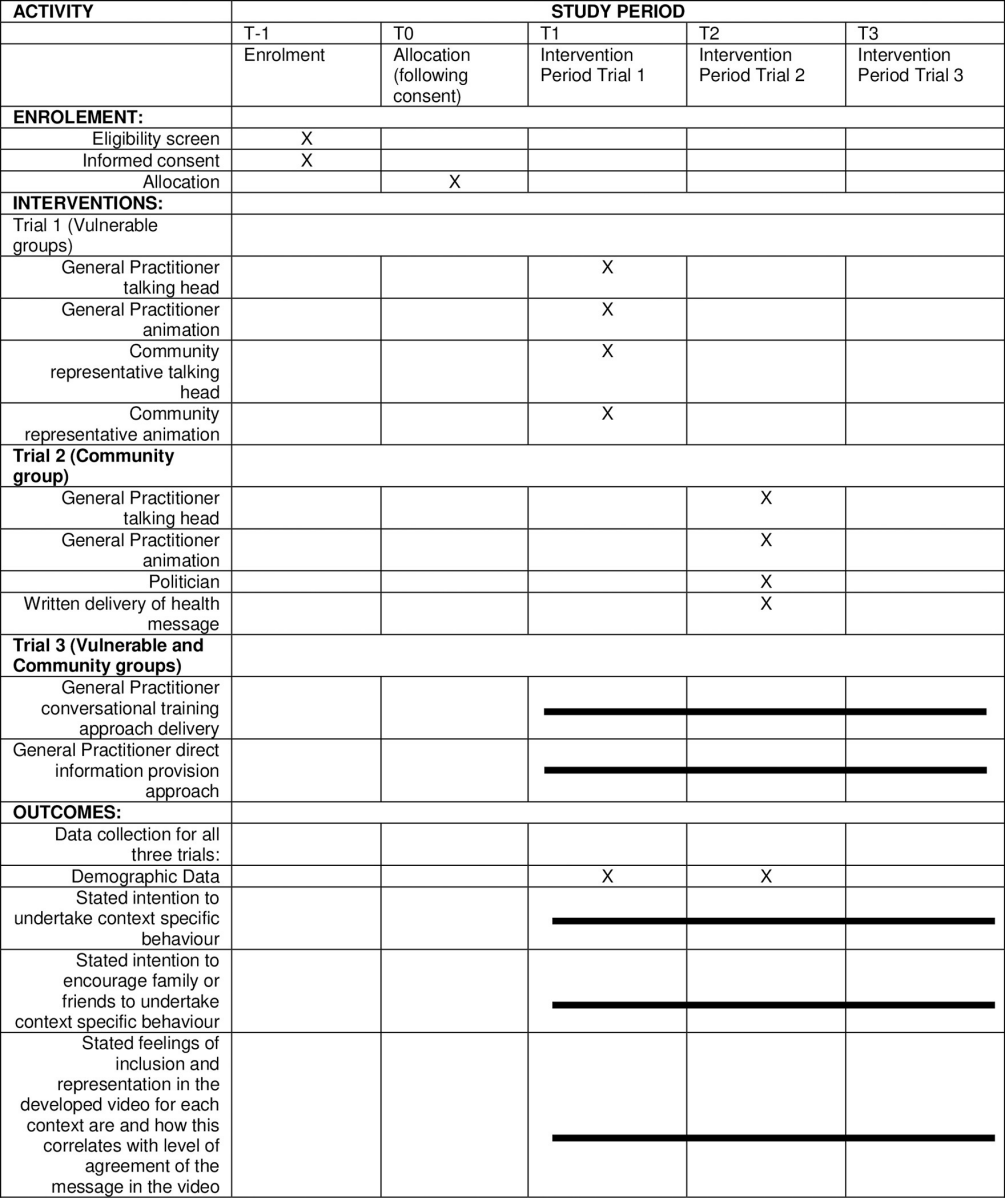
SPIRIT flow diagram: Schedule of enrolment, interventions, and assessment procedures.

**Fig 2:**
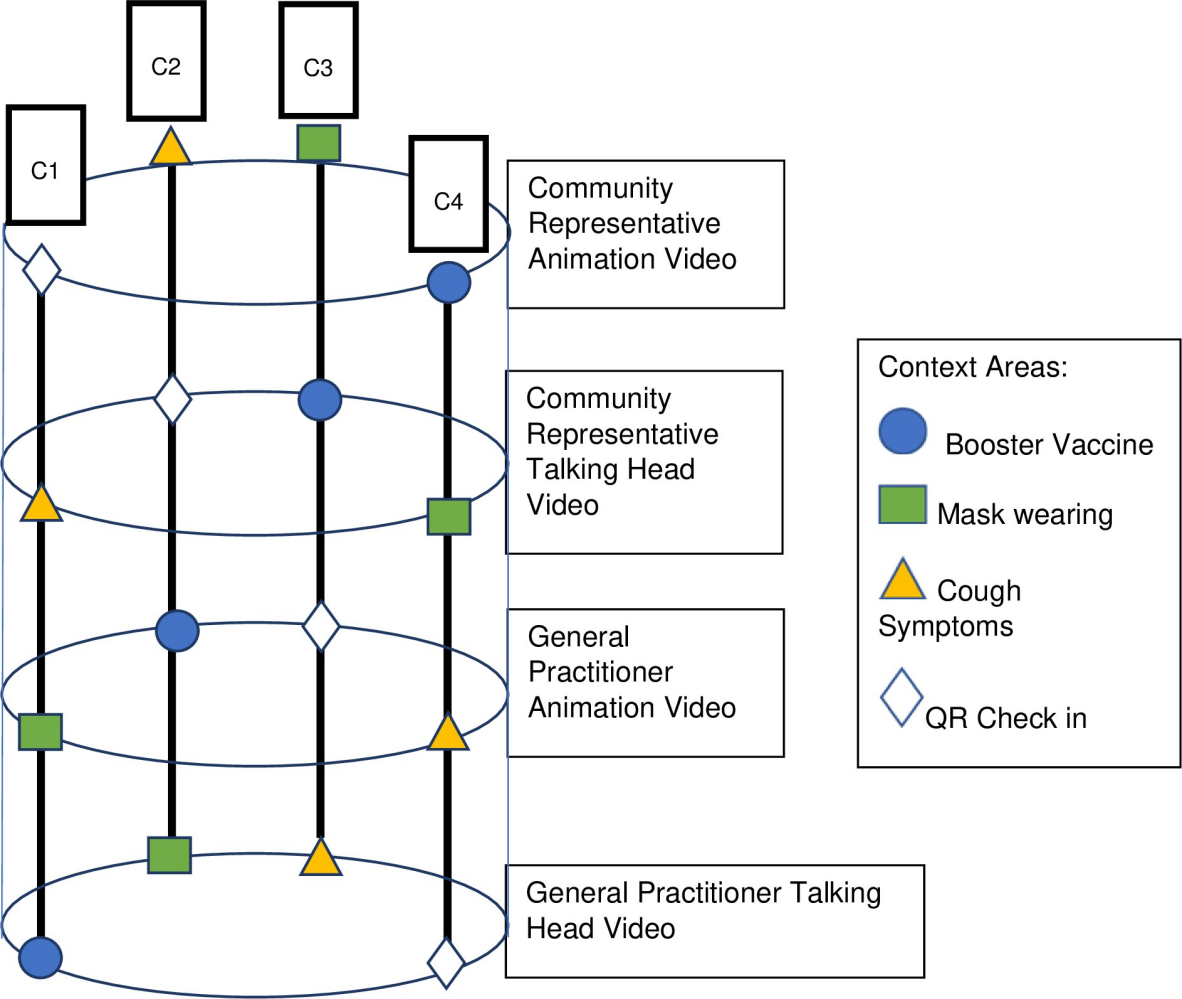
Visual representation of the helix randomised control trial design for Trial 1. C1 denotes combination 1, C2 denotes combination 2, C3 denotes combination 3, C4 denotes combination 4. Individual participants are exposed to the combinations of intervention level / context areas within a vertical combination column.

**Fig 3:**
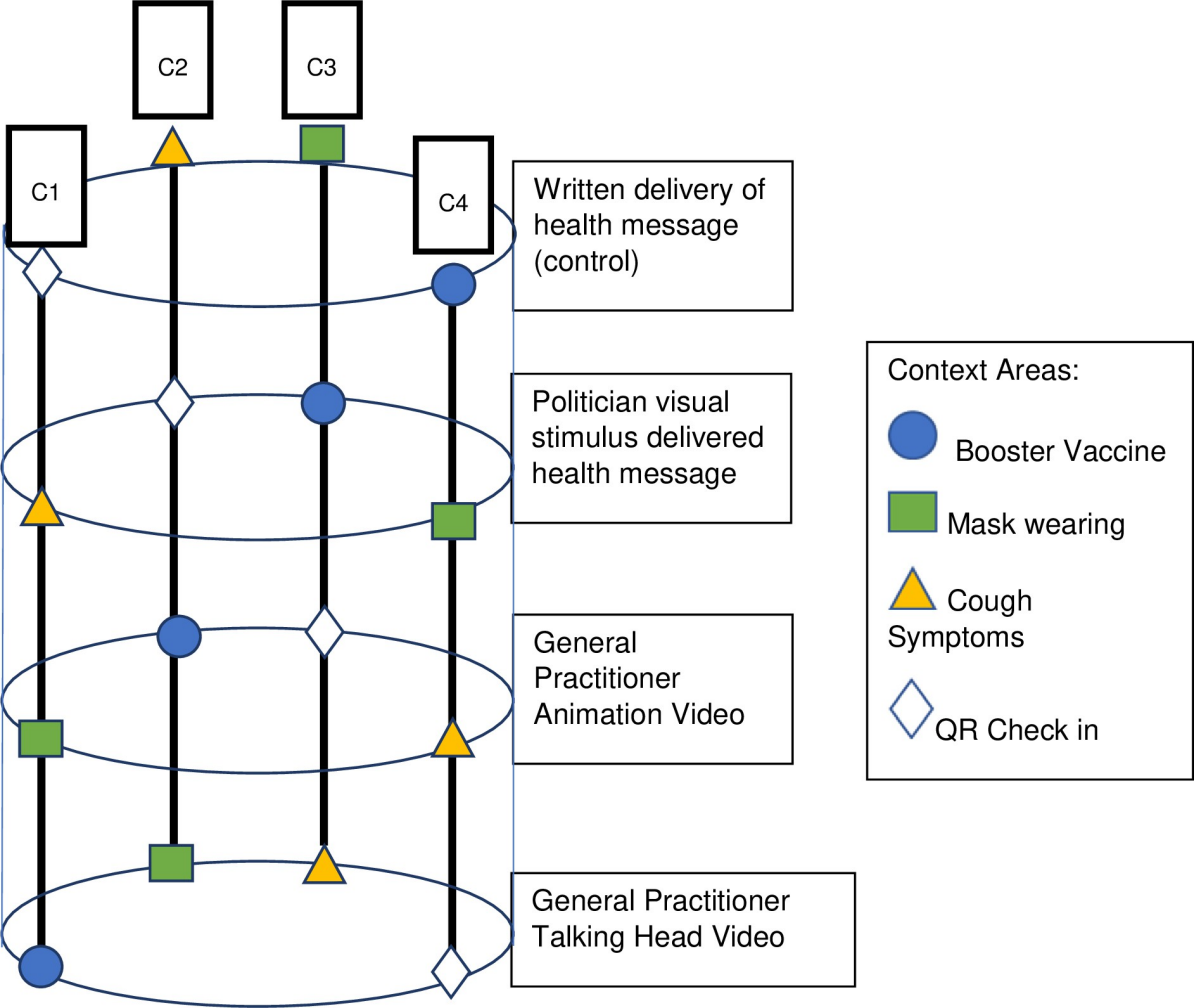
Visual representation of the helix randomised control trial design for Trial 2. C1 denotes combination 1, C2 denotes combination 2, C3 denotes combination 3, C4 denotes combination 4. Individual participants are exposed to the combinations of intervention level / context areas within a vertical combination column.

**Fig 4:**
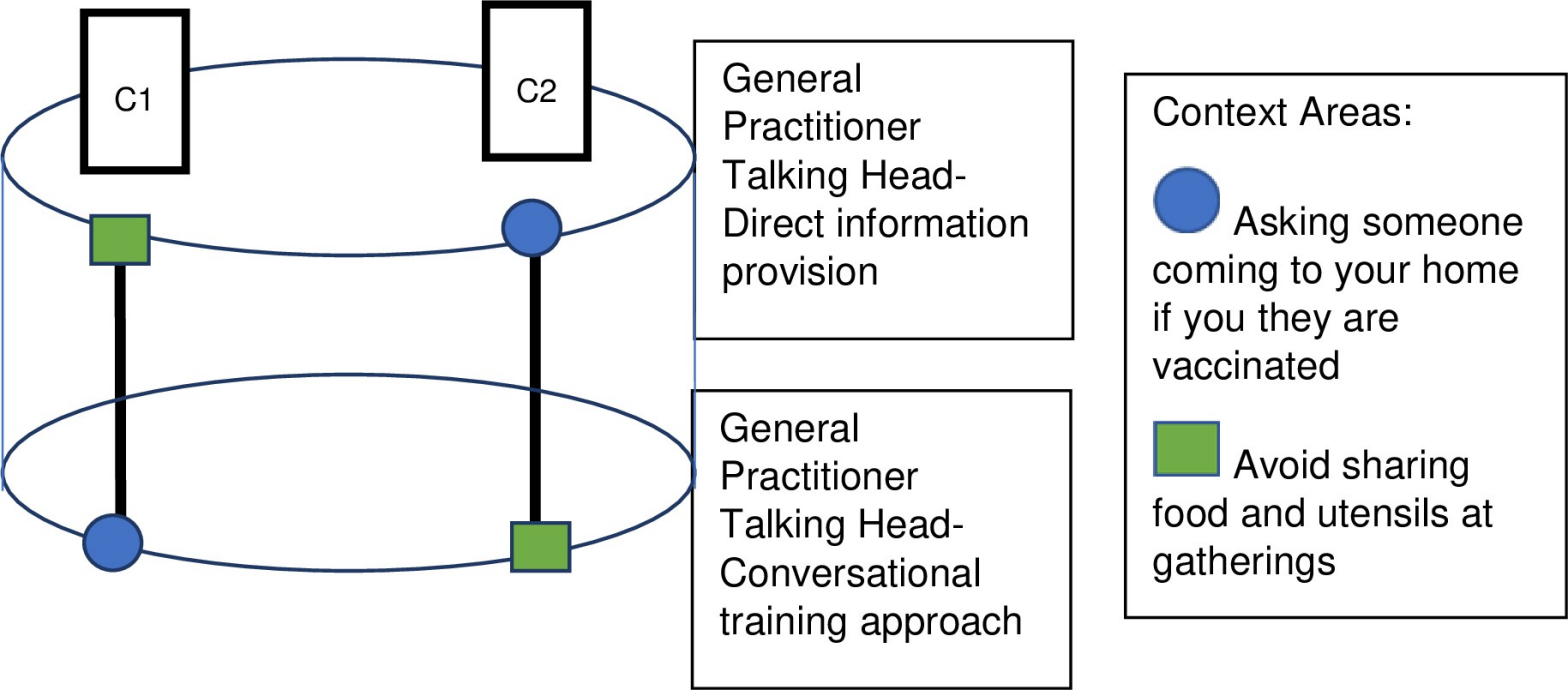
Visual representation of the helix randomised control trial design for Trial 3. C1 denotes combination 1, C2 denotes combination 2, Individual participants are exposed to the combinations of intervention level / context areas within a vertical combination column.

The Standard Protocol Items: Recommendations for Interventional Trials (SPIRIT) flow diagram schedule of enrolment, interventions and assessment procedures is provided in Fig 1 [38]. We will conduct three concurrent ‘helix’ randomised controlled trials (Figs 2–4), as approved by Monash University Human Research Ethics Committee on the 8^th^ April 2022 (HREC 30698). The Helix design is a counterbalanced, randomised controlled trial that involves allocating participants to each level of an intervention, but spread across different context areas. The outcome measure is repeated for each intervention level / context area exposure, allowing for a pooled analysis with repeated measures within participants. This enhances statistical power of an investigation while minimising the risk of intervention-level contamination by separating them across the different context areas.

In trial 1, we will combine the ‘helix’ approach with factorial allocation of intervention levels. In all trials we will be using Latin-square sequencing of the intervention levels to minimise the risk of order effects from potentially biasing the contrasts between intervention levels. Participants will be exposed to context areas in a set order (meaning that order effects could impact contrasts between them) as comparisons between contexts are not of primary interest in these investigations.

### Participants and setting

This study will include a sample of the general Australian community as well as six subgroup population groups. Trial 1 will only include participants from the six vulnerable population groups, Trial 2 will only include participants from the general Australian community, and Trial 3 will include participants from all six vulnerable subgroups as well as the general Australian community.

These communities can be argued to be vulnerable to infection, transmission and experiencing negative health and other outcomes during the COVID-19 pandemic on the basis of their pre-existing lower health equity due to socioeconomic factors, increased risk of COVID-19 exposure, cultural factors, need for support for decision making due to intellectual or cognitive changes and/or augmentative or alternative communication methods or challenges experienced in communicating (Table 1) [10, 39, 40].

**Table 1:** Project population subgroups and their associated COVID-19 risk factors & communication challenges.

COVID-19 risk factors & communication challenges	Population subgroup					
	Aboriginal & Torres Strait Islander peoples	People living with disability & their carers	Refugee & asylum seekers	Deaf, hard of hearing people	Aged care workers	Street-based sex workers
Higher exposure risk		**Applicable** [12, 41] due to personal care tasks			**Applicable** due to work	**Applicable** [6, 7] due to work
Higher rates of health conditions & need for health services	**Applicable** [12, 41]	**Applicable** [12, 41]	**Applicable** [42]			**Applicable** [7]
Difficulty accessing health care	**Applicable** [41]		**Applicable** [42]			**Applicable** [6, 7]
Higher rates of limited English proficiency			**Applicable**		**Applicable**	
Lower rates of digital literacy and access to the Internet or Internet-enabled devices		**Applicable** [43, 44]				
Limited accessible COVID-19 information		**Applicable** [12]		**Applicable** [45]		
Loss of educational support and access		**Applicable**	**Applicable**	**Applicable** [46]		
Different cultural understandings of illness	**Applicable**		**Applicable**			
Lower SES	**Applicable** [47]	**Applicable** [12]	**Applicable** [42]			**Applicable** [7]
Higher rates of contract, casual or precarious work		**Applicable** [48]			**Applicable**	**Applicable** [6, 7]
Housing situation	**Applicable** [47] Larger, multi-family households		**Applicable** Larger, multi-family households			**Applicable** [6] Lack of appropriate housing

Participant groups in this study will be:

1. People who are currently living with a disability or a carer of someone who has a disability.
2. Aged care workers.
3. Refugee and asylum seekers with limited English proficiency whose first language is either Dari or Sinhalese. We limited this group to two non-English speaking communities as a project feasibility measure. None of the people in this cohort have Australian citizenship.
4. Deaf and hard of hearing people who are Auslan users.
5. Aboriginal and Torres Strait Islander peoples.
6. Street-based sex workers.
7. General Australian community.

People not currently living in Australia or who are under the age of 18 years will be excluded from this research.

Participants across all trials will observe the videos either in their own home (due to the online nature of the surveys), or at a partner organisation service provider location relative to their vulnerable population subgroup (if applicable). For example, participants in the Aboriginal Torres Strait Islander subgroup, may complete the survey at a common community meeting place.

### Interventions

#### Trial 1 (vulnerable groups only)

This trial will involve factorial allocation of two intervention factors each with two levels (2x2 factorial) across four context areas. These factors are:

Factor 1: Who is providing the message (general practitioner versus community representative).
Factor 2: How is the message being delivered (animation versus talking head).

Both general practitioners and community representatives will be selected due to their high level of perceived trust in vulnerable subgroup communities, making them a viable potential deliverer of messages in real life. However, from a public health messaging perspective, a general practitioner can be used to develop a message that may potentially impact a range of vulnerable groups quickly in a pandemic context, whereas it takes more time and greater cost to produce messages delivered by specific vulnerable community representatives as each vulnerable community requires its own representative and own message to be produced. The talking head versus animation intervention approaches were selected as both are commonly used, yet are competing visual imagery approaches in public health messaging.

The project team contains a general practitioner who will perform this role for materials production. Scripting of the intervention messaging will be consistent with publicly available messages that have been forwarded by government and will be held as constant as feasible between the intervention levels. Community representatives will be specifically selected for each subgroup population based on their established leadership. They will also be invited to co-design messaging to ensure it is appropriate for the specific vulnerable population. Two of the vulnerable subgroup populations will require translation (of the written survey material) and interpreting (of the video content). This will be English to Dari and Sinhalese translation and interpretation for refugee and asylum seeker subgroup who have limited English proficiency and English to Auslan interpreting for the deaf and hard of hearing subgroup. During the video production phase, we will aim to ensure the consistency of ‘who’ across the general practitioner and community representative videos by purposely using a community representative who is recognisable and familiar to the participants. For these groups, interpretation of the general practitioner videos will be completed by a different person (who will not be as recognisable or familiar to the participants). For the refugee and asylum seeker subgroup, the general practitioner interpreter will not be visible on screen and provide spoken interpretation only, into either Dari or Sinhalese (English subtitles will remain on screen for both community representative and general practitioner videos). For the deaf and hard of hearing subgroup, the interpreter will remain on screen providing Auslan interpretation, with English subtitles also available for all videos except the community representative videos.

These contexts across which the intervention levels will be provided will be:

1. Having a booster vaccine
2. Wearing a mask outdoors when you can’t physically distance
3. Staying at home when you have a new cough symptom
4. Using QR check in codes when required

Video-based public health messages will be constructed to be up to 2 minutes in duration which we considered to be the upper limit of time used for public health messaging.

#### Trial 2 (community group only)

This trial includes four intervention arms, across the same four COVID-19 health messaging context areas as Trial 1.

The study arms include:

1. General practitioner talking head visual stimulus delivered health message (same materials as Trial 1).
2. Animated general practitioner visual stimulus delivered health message (same materials as Trial 1).
3. Politician visual stimulus delivered health message
4. Written delivery of health message (control)

Each participant will be exposed to each of the four intervention arms (control written provision of health message, general practitioner talking head, general practitioner ’animation’, politician delivered message). The content of the messaging included in each of these four intervention arms will be held as constant as possible.

We did not use the same factorial design as we used in Trial 1 for the Trial 2 general community sample as we considered that there was no truly representative person we could use for the ‘community representative’ intervention level. We pivoted in our planning to consider the impact of general practitioners (high levels of trust) [49] to politicians (lower levels of trust) [19, 22] under the reasoning that politicians became the ‘face’ of public health messaging during the COVID-19 pandemic in Australia when others (such as general practitioners) could have been, and we desired to understand the potential consequences of this. The politician videos will be constructed from specific edits of existing publicly available footage from politicians at both state and federal levels and on different sides of the ‘political divide’ in Australia to try to limit the impact that political party preferences might have on the comparison between politicians and general practitioners for message delivery.

Like Trial 1, Trial 2 will enable comparison of animated versus talking head visual representations by contrasting Trial 2 intervention arms 1 and 2 as these will be the same videos as used in Trial 1. It will also allow examination of the moderating effect that population subgrouping (general community versus vulnerable groups) might have on the relationship between the visual representation factor and study outcomes.

The written materials will be based as closely as possible to the scripts of the videos used so as to hold content as being consistent in these contrasts. This arm (Arm 4) was selected to serve as a generic control that allows for comparison of the different video-based presentation approaches to a written information approach, that might resemble written information available on a government website.

#### Trial 3 (all participants)

Trial 3 will include two intervention arms across two different context areas related to COVID-19 health. The two arms of the study will be:

1. Conversational training approach provided by a general practitioner (talking head).
2. Direct information provision provided by a general practitioner (talking head).

The arms of study differ by the language, content and tone. The conversational training approach provides a health message in a peer-to-peer fashion that is expressed in a relaxed conversational tone. The direct information provision arm, provides a health message that is direct and didactic in style, and does not provide suggested statements that people could say in a discussion with another person on this topic.

The two context areas will be:

1. Encouraging people who have workers coming to visit them at home to ask them if they are vaccinated or not.
2. Encouraging people to avoid sharing food, utensils and other items at gatherings.

### Outcomes

#### Primary outcome 1: Stated intention to undertake a context specific behaviour

Behavioural intentions are commonly used as proxy measures for subsequent behaviours in contexts where valid measurement of subsequent behaviours is unfeasible [50]. Aligned with this, participants will be asked their intention to undertake a context specific behaviour after viewing the intervention. Responses will be scaled using a 5-point Likert Scale (‘much less inclined’, ‘less inclined’, ‘about the same’, ‘more inclined’ and ‘much more inclined’).

#### Primary outcome 2: Stated intention to encourage family or friends to undertake a context specific behaviour

Participants will be asked their intention to encourage family or friends to undertake a context specific behaviour after viewing the intervention. Responses will be scaled using a 5-point Likert Scale (‘much less inclined’, ‘less inclined’, ‘about the same’, ‘more inclined’ and ‘much more inclined’). Levels of trust in family or friends has been shown to be similar to other sources regarding COVID-19 information.(23) This level of trust, combined with the high levels of opportunity for conversations to take place between family and friends regarding public health measures makes this arguably an important outcome to influence.

#### Secondary outcome 1: Stated feelings of inclusion or representation in the developed video and how they correlate to level of agreement of the message in the video

For this outcome, we are aiming to understand how represented the participants feel in each of the intervention videos, and what impact this has on the level of agreement to the message in the video. Immediately after viewing each video, participants will be asked to indicate their level of agreement using a 5-point Likert Scale (’strongly disagree’, ’disagree’, neither agree nor disagree’, ’agree’, ’strongly agree’).

Once all interventions have been viewed, participants will then be asked if being strongly represented in a health message videos impacts the extent to which they are more inclined to agree with the message in a video public health message using a 5-point Likert Scale (’strongly disagree’, ’disagree’, neither agree nor disagree’, ’agree’, ’strongly agree’).

### Procedure

Vulnerable subgroup populations will be recruited from partner organisations listed in Table 2. An advertisement via email, in person or via the partner organisation social media pages will be used, explaining the basic details of the project and what is required. A link to the explanatory statement and survey will also be included in this advertisement and participants will be able to complete the survey independently in their own time, with assistance from a family member or carer, or with assistance from a member of the corresponding partner organisation team if completing the survey on site. All information (explanatory statements, survey questions and video information) will be captioned, translated, interpreted or adapted accordingly to the communication profiles of the different vulnerable subgroup populations. This includes Auslan for the deaf and hard of hearing group, and Dari and Sinhalese for the refugee and asylum-seeker group. The general community group will be recruited online using social media platforms (i.e. Facebook and Twitter).

**Table 2:** Partner organisations related to each subgroup population.

Vulnerable Subgroup	Partner Organisation
**People living with a disability and their carers**	Able Australia, Wallara and Yooralla
**Aged care workers**	Donwood Community Aged Care Services
**Refugee and asylum seekers**	enliven
**Deaf, hard of hearing people**	Expression Australia and Able Australia
**Aboriginal and Torres Strait Islander peoples**	Peninsula Health
**Street-based sex workers**	St Kilda Gatehouse
**General Australian community**	Not applicable.

Randomisation will occur automatically via the inbuilt randomisation function on the Qualtrics data collection platform (Qualtrics, Provo, UT, USA). Therefore, random allocation will be fully concealed from participants and investigators involved in recruitment and data collection.

### Study timeline

Participants will be able to access the relevant survey (to their subgroup) leading to either Trial 1 then Trial 3 (combined) or Trial 2 then Trial 3 (combined) independently or with assistance from the corresponding Partner Organisation. By following the link to the corresponding survey for each subgroup population, participants will have access to the explanatory statement including inclusion and exclusion criteria. From there, participants can choose to participate and confirm they adhere to the inclusion criteria. The data collection and survey builder software Qualtrics (Qualtrics, Provo, UT, USA) will automatically, randomly allocate participants into one of four potential combinations of allocation using a Latin Square approach for both Trial 1 and Trial 2, (minimising study level order effects), and will again randomly assign to one of two possible combinations for Trial 3. Data will be extracted for analysis purposes with no follow-up period required. Refer to Fig 1 for an overview of the study timeline.

### Data management

Information relating to the participation in the trial will not be available to any persons outside the study team. De-identified aggregate data from this research will be shared publicly only in peer reviewed journals, conferences and reports back to the participants and partner organisations (where appropriate). De-identified results data can be made available upon request to study investigators.

### Analysis

The comparisons we will undertake across the three trials will be:

#### Trial 1

1. Main effect of who is providing the message (General Practitioner versus community representative).
2. Main effect of visual stimulus (talking head versus animation).
3. Interaction effect between these two main effects.
4. Simple effects if interaction effect is significant.

#### Trial 2

1. General practitioner talking head visual stimulus delivered health message versus animated general practitioner visual stimulus delivered health message.
2. Politician visual stimulus delivered health message versus general practitioner talking head visual stimulus delivered health message.
3. Written delivery of health message (control) versus all other intervention types (general practitioner talking head, general practitioner animation and politician delivered health message).

#### Trial 3

1. The effectiveness of health information provision delivered through a conversational training approach by a general practitioner versus through a direct information provision approach by a general practitioner.

Comparisons will not be made between context areas as part of the primary analysis. However, we will investigate interaction effects of the different intervention comparisons across the different context areas and population subgroups so that we can understand whether certain interventions work better in certain contexts and populations.

Linear mixed model analyses will be used treating the intervention levels as fixed factors and participants as random factors. Context will be ignored when investigating intervention main effects as it will be balanced across the different intervention levels.

### Sample size

The repeated-measures, latin square design creates an analysis paradigm comparable to a paired-sample comparison of proportions. The least statistical power generated through the helix design arises when there is a simple contrast between two levels of an intervention within a singular vulnerable group category. Power analysis for this context can be approximated by a power analysis conventionally performed for McNemar’s Test, though we will be using linear mixed models for our analyses. A sample size of 36 participants per subgroup will provide 80% to detect a difference in proportions of respondents being “more inclined” or “much more inclined” with a primary outcome statement from 0.2 to 0.4. This assumes a correlation between the outcome proportions of (0.61) and a two-tailed alpha of 0.05. We aim to recruit 40 participants per vulnerable subgroup to allow for ~10% partial completion of the full survey. The study is conducted in a single session so there is no further allowance for loss to follow-up (this is irrelevant in our context). No maximum sample size will be set for the community sample given our online recruitment approach and short time-frame for data collection negating the need to cease the trial “early” due to important findings being identified.

## Discussion

This research will involve collaborative input from partner organisations relating to each of the six vulnerable subgroup populations throughout. Previous research into health promotion for vulnerable groups has argued the positive benefits of co-creating health communication strategies with participants of the relevant cultures and communities from the outset and throughout the process [51, 52]. Therefore, all video materials will be co-designed by a steering committee comprised of the research team, representatives of multiple relevant community organisations and government representatives. This aims to ensure that findings from this research will be relevant and culturally appropriate for each vulnerable subgroup population.

There are potential economic benefits to investigating the different approaches to delivering public health messaging. This is particularly relevant given the negative global economic consequences of COVID-19 [53]. For example, if a message from a trusted medical expert has the same impact on all vulnerable groups as does a message delivered by individual community leaders from each group, then that could lead to less need for the production of multiple pieces of media by multiple individuals, thus potentially reducing overall cost. Furthermore, the cost of producing one animation that can be repeated across all individual population groups would be more cost-effective than having to produce individual pieces of media for each group, particularly if they are found to have a similar effect on behavioural intentions. However, where groups may require a message to be translated into another language, pre-recorded videos can impair the quality and accessibility of the translation because they force the translators to work within the time restraints of the existing visuals, rather than allowing the message to take as long as it needs in the new language [54].

There has been a wealth of information about COVID-19 that has been accessible to the community. The Director-General of the World Health Organisation referred to this as an ‘infodemic’ [55]. This appears to have led to the spread of misinformation from various sources [32]. Previous research has suggested that incorrect claims can propagate faster than correct information on social media [16, 56]. Therefore, there is a need for clear, trustworthy public health messaging that can also spread quickly and that can potentially counter-act the spread of misinformation.

The findings of this research will aim to inform the design and delivery of future COVID-19 and of future public health messaging in general, particularly that directed at vulnerable communities. These findings aim to lead to the production of more impactful and culturally relevant health communication strategies, thus leading to improved health outcomes for vulnerable communities, while potentially reducing costs of communication material production for governments and relevant organisations.

### Limitations

This study is limited in its reliance on having behavioural intentions as the primary end-point instead of actual behaviours and health outcomes. Behavioural intentions do not always correspond with subsequent actions [57], though we do consider them to be the most appropriate primary outcome for the present study as not all participants will be exposed to the situations where the specific behaviours may be required (e.g. having a tradesperson come to their house) within a reasonable follow-up timeframe. Difficulty eliciting honesty through self-report given previous government restrictions and regulations that may still be perceived as being imposed is another limitation to understanding behaviour in this context. Other limitations of this study include the ever-changing nature of the COVID-19 global pandemic, variation in government preventative health measures, and that the community’s attitudes and knowledge to particular public health messages may change quickly. Finally, although efficient in both research timing and study participant access, recruiting the majority of participants online means, some people who are not familiar with the use of technology, or who have limited access to technology or the internet, might be inadvertently excluded from participating.

### Protocol amendments

Amendments to the study protocol will require approval from the Monash University Human Research Ethics Committee as well as study investigators. Any amendments will be communicated via updates through the applicable trial registration (through ANZCTR), and reported in any published manuscript associated with the study.

## Supporting information

S1 File SEPTRE SPIRIT checklist. (DOC)

S2 File MUHREC approved. (PDF)
