# Supplementary material for: Effectiveness of tailored COVID-19 messages for vulnerable Australians: A study protocol
Source: PLoS One. 2023 Jan 27;18(1):e0280865. doi: 10.1371/journal.pone.0280865 (PMC9882702; doi:10.1371/journal.pone.0280865)
Supplement: S2 File — (PDF) [file pone.0280865.s002.pdf]

[redacted]

# Human Ethics Application Form

### Instructions

Monash University Human Research Ethics Committee (MUHREC) ensures that research at Monash University complies with the National Statement on Ethical Conduct in Human Research 2007. For further information about MUHREC including upcoming **meeting dates** click here.

**Which form should I use?**

Refer to the table below for information on which form to complete and contact the human ethics office on 99052052 if you are not sure which form to use.

| Human Ethics Application form | Human Ethics Other HREC |
|---|---|
| – When Monash staff or students are conducting the research; OR<br>– When the funds are administered through Monash; OR<br>– When you have approval to access data from a Scientific Research Committee but do not have HREC approval. | When Monash staff or students are working on projects that have current approval from another HREC and the project is ongoing at the other institution. |
| When researchers are transferring to Monash and have current ethics approval from their Australian institution. | When Monash staff are recruiting hospital patients or staff or accessing hospital data. Approval from the hospital HREC must be obtained first and then the project can be registered using this form. |

**How to navigate ERM**

- Move through the form using the **'Next/Previous'** buttons in the action panel
- The **'Navigate'** button takes you back to the form overview
- The **'Completeness Check'** button will alert you to any mandatory questions you need to complete before you are able to submit the form
- Click the blue **'Info'** icon on the right-hand side for guidance when responding to questions
- Click **'Submit'** to send your application to MUHREC for review - MUHREC cannot see your application until it is submitted
- You will receive an **email receipt** once you have successfully submitted your application

The **Chief Investigator (CI) listed in A2 must be the main Monash Supervisor** of this project. If you have created the form, but are not the CI, your details will be incorrectly listed in Question A2. To proceed, complete the form listing yourself under the appropriate investigator subheading (e.g. student), then **transfer** the project to the CI. Once the CI accepts the transfer, their details will automatically override your details in Question A2. The CI should then **share** the project with you and **submit** the application. Refer to the Ethics Review Manager (ERM) User Guide for instructions on transferring and sharing projects.

**Access to Monash ERM** is restricted to Monash staff and students. You can add external co-investigators at question A4 but you cannot share the project with them and you will need to send them a PDF of the application instead.

**If you cannot see the submit button**, check for a **red banner** at the top of this page informing you of a recent update - the CI (project owner) must log in and click on the banner to update the form. If there is no banner, and you are not the CI, click the '**Collaborators**' button to see if you have been granted permission to submit - if you do not have permission, the CI will need to click submit.

## Checklist

**Select at least one option on this page. Your response determines if your application is low risk, or requires review at a MUHREC meeting.**

**This project aims to specifically recruit from the following participant groups:**

- [x] Aboriginal and Torres Strait Islander participants
- [ ] Women who are pregnant and the human fetus
- [ ] People with a cognitive impairment, an intellectual disability, or a mental illness, e.g. brain injury, dementia, ADHD, ASD etc.
- [ ] People considered to be a forensic patient, an involuntary patient or a security patient
- [x] People with impaired capacity for communication
- [ ] Prisoners or people on parole*
- [ ] Children who are Wards of State*
- [ ] People highly dependent on medical care including a person who is unconscious*
- [ ] Military personnel and / or veterans*
- [ ] Victoria Police personnel*
- [ ] Patients, staff or data from hospitals that have an ethics committee*

**AND / OR this project involves the following procedures:**

- [ ] Use of identifiable / coded health information or biospecimens without consent e.g. medical records, data linkage
- [ ] Any physical / psychological/social/economic or legal risks greater than inconvenience or discomfort, in either the short or long term, resulting from participation, or use of data in this project
- [ ] Interventions or therapies e.g. administration of drugs, use of device, clinical or psychological treatments
- [ ] Sensitive / contentious issues e.g. suicide, eating disorders, body image, trauma, violence, abortion
- [ ] Radioactive substances / Ionising radiation e.g. DXA, X-ray
- [ ] Intends to study / expose illegal activity
- [ ] Human genetics
- [ ] Derivation of human embryonic stem cells
- [ ] Assisted reproductive technology
- [ ] Deception of participants, concealment or covert observation

**OR this project DOES NOT involve any of the participants or procedures listed above**

*Selecting this option will uncheck any boxes selected above*

- [ ] None of the above

## Application Details

### A1 Project Title

Uptake and impact of Government recommendations about COVID-19 (coronavirus)- Stage 3

### A2 Chief Investigator *(Monash staff or adjunct only, not affiliate)*

- **For student research projects, the main Monash supervisor should be listed as the Chief Investigator (CI).**
- The information below has been retrieved from the profile of the user creating this application and is not editable. If you are listed here but you are not the CI, you must ensure that you are listed under the appropriate investigator subheading (e.g. student), then transfer the project to the CI. The CI should then share the project with you and submit the application. Refer to the ERM User Guide for instructions on how to transfer a project.

| Title | First Name | Surname |
|---|---|---|
| Ms | Megan | Jepson |

| | |
|---|---|
| Organisation | Monash University (Staff) |
| Department | Department of Paramedicine |
| Faculty | Faculty of Medicine Nursing & Health Sci |
| Campus | Monash Peninsula |
| Email | Megan.Jepson@monash.edu |

### A3 Please list who we should contact if there are questions regarding this project

| Full Name | Preferred phone number |
|---|---|
| Terry Haines | 99029409 |

## Other Investigators

- **If Monash staff or students do not appear in the search field, they will need to log into ERM once in order to activate their profile. You will then be able to add them and share or transfer applications to them.**
- **Monash investigator details cannot be edited - the information is retrieved from the Monash University Identity Management System.**

### A4 Other investigators involved in this research include:

- [x] Co-investigator
- [ ] Project Coordinator
- [x] Research Assistant
- [ ] Student
- [x] External (Non-Monash) Investigator
- [ ] None

**Please list all Co-investigators involved.**

*To give this co-investigator access to the application, click 'Roles' in the Actions panel on the left, enter their email address and select the required access in the dropdown list.*

| Title | First Name | Surname |
|---|---|---|
| Professor | Terry | Haines |

Department: Sch of Primary & Allied Health Care

Faculty: Faculty of Medicine Nursing & Health Sci

**Please list all Co-investigators involved.**

Email: Terry.Haines@monash.edu

*To give this co-investigator access to the application, click 'Roles' in the Actions panel on the left, enter their email address and select the required access in the dropdown list.*

| Title | First Name | Surname |
|---|---|---|
| Professor | Grant | Russell |

Department: Department of General Practice

Faculty: Faculty of Medicine Nursing & Health Sci

**Please list all Co-investigators involved.**

Email: Grant.Russell@monash.edu

*To give this co-investigator access to the application, click 'Roles' in the Actions panel on the left, enter their email address and select the required access in the dropdown list.*

| Title | First Name | Surname |
|---|---|---|
| Professor | Keith | Hill |

Department: Sch of Primary & Allied Health Care

Faculty: Faculty of Medicine Nursing & Health Sci

**Please list all Co-investigators involved.**

Email: Keith.Hill@monash.edu

*To give this co-investigator access to the application, click 'Roles' in the Actions panel on the left, enter their email address and select the required access in the dropdown list.*

| Title | First Name | Surname |
|---|---|---|
| Assoc Professor | Cylie | Williams |

Department: Sch of Primary & Allied Health Care

Faculty: Faculty of Medicine Nursing & Health Sci

**Please list all Co-investigators involved.**

Email: Cylie.Williams@monash.edu

*To give this co-investigator access to the application, click 'Roles' in the Actions panel on the left, enter their email address and select the required access in the dropdown list.*

| Title | First Name | Surname |
|---|---|---|

| | | |
|---|---|---|
| Assoc Professor | Elizabeth | Callaway |

| | |
|---|---|
| Department | Occupational Therapy |
| Faculty | Faculty of Medicine Nursing & Health Sci |

**Please list all Co-investigators involved.**

Email: Libby.Callaway@monash.edu

*To give this co-investigator access to the application, click 'Roles' in the Actions panel on the left, enter their email address and select the required access in the dropdown list.*

| Title | First Name | Surname |
|---|---|---|
| Dr | Jim | Hlavac |

Department: Translation Studies

Faculty: Faculty of Arts

**Please list all Co-investigators involved.**

Email: Jim.Hlavac@monash.edu

*To give this co-investigator access to the application, click 'Roles' in the Actions panel on the left, enter their email address and select the required access in the dropdown list.*

| Title | First Name | Surname |
|---|---|---|
| Assoc Professor | Louisa | Willoughby |

Department: Linguistics

Faculty: Faculty of Arts

**Please list all Co-investigators involved.**

Email: Louisa.Willoughby@monash.edu

*To give this co-investigator access to the application, click 'Roles' in the Actions panel on the left, enter their email address and select the required access in the dropdown list.*

| Title | First Name | Surname |
|---|---|---|
| Dr | Katrina | Long |

Department: Sch of Primary & Allied Health Care

Faculty: Faculty of Medicine Nursing & Health Sci

**Please list all Co-investigators involved.**

Email: Katrina.Long@monash.edu

*To give this co-investigator access to the application, click 'Roles' in the Actions panel on the left, enter their email address and select the required access in the dropdown list.*

| Title | First Name | Surname |
|---|---|---|
| Dr | Matt | Loads |

Department: Bachelor of Media Communications

Faculty: Faculty of Arts

Email: Matthew.Loads@monash.edu

**Please list all research assistants involved.**

*To give this research assistant access to the application, click 'Roles' in the Actions panel on the left, enter their email address and select the required access in the dropdown list.*

| Title | First Name | Surname |
|---|---|---|
| Dr | Lauren | Robins |

| | |
|---|---|
| Department | Sch of Primary & Allied Health Care |
| Faculty | Faculty of Medicine Nursing & Health Sci |
| Email | Lauren.Robins@monash.edu |

**Please list all research assistants involved.**

*To give this research assistant access to the application, click 'Roles' in the Actions panel on the left, enter their email address and select the required access in the dropdown list.*

| Title | First Name | Surname |
|---|---|---|
| Ms | Megan | Jepson |

| | |
|---|---|
| Department | Department of Paramedicine |
| Faculty | Faculty of Medicine Nursing & Health Sci |
| Email | Megan.Jepson@monash.edu |

**Please list all research assistants involved.**

*To give this research assistant access to the application, click 'Roles' in the Actions panel on the left, enter their email address and select the required access in the dropdown list.*

| Title | First Name | Surname |
|---|---|---|
| Mr | Nathan | Williams |

| | |
|---|---|
| Department | Sch of Primary & Allied Health Care |
| Faculty | Faculty of Medicine Nursing & Health Sci |
| Email | Nathan.Williams@monash.edu |

**Please list all research assistants involved.**

*To give this research assistant access to the application, click 'Roles' in the Actions panel on the left, enter their email address and select the required access in the dropdown list.*

| Title | First Name | Surname |
| --- | --- | --- |
| Dr | Glen | Whittaker |

| | |
| --- | --- |
| Department | Sch of Primary & Allied Health Care |
| Faculty | Faculty of Medicine Nursing & Health Sci |
| Email | Glen.Whittaker@monash.edu |

**Please list all research assistants involved.**

*To give this research assistant access to the application, click 'Roles' in the Actions panel on the left, enter their email address and select the required access in the dropdown list.*

| Title | First Name | Surname |
| --- | --- | --- |
| Dr | Angel | Lee |

| | |
| --- | --- |
| Department | Sch of Primary & Allied Health Care |
| Faculty | Faculty of Medicine Nursing & Health Sci |
| Email | Angel.Lee@monash.edu |

**Please list all research assistants involved.**

*To give this research assistant access to the application, click 'Roles' in the Actions panel on the left, enter their email address and select the required access in the dropdown list.*

| Title | First Name | Surname |
| --- | --- | --- |
| Dr | Em | Bould |

| | |
| --- | --- |
| Department | Occupational Therapy |
| Faculty | Faculty of Medicine Nursing & Health Sci |
| Email | Em.Bould@monash.edu |

**Please list all research assistants involved.**

*To give this research assistant access to the application, click 'Roles' in the Actions panel on the left, enter their email address and select the required access in the dropdown list.*

| Title | First Name | Surname |
|---|---|---|
| Ms | Suzanne | Grasso |

Department: Communications & Community

Faculty: Chief Operating Officer & Senior VP

Email: Suzanne.Grasso1@monash.edu

**Please list the external (Non-Monash) investigators involved.**

*You cannot search the Monash directory for non-Monash investigators or share the project with them as access to ERM is restricted to Monash staff and students. You need to enter the details below manually.*

| Title | First Name | Surname |
|---|---|---|
| Ms | Rachel | Lennon |

Organisation: Australian and New Zealand College of Anaesthetists

Email: rachellennon78@gmail.com

**Please list the external (Non-Monash) investigators involved.**

*You cannot search the Monash directory for non-Monash investigators or share the project with them as access to ERM is restricted to Monash staff and students. You need to enter the details below manually.*

| Title | First Name | Surname |
|---|---|---|
| Mr | Dwayne | Pearce |

Organisation: Peninsula Health

Email: dwaynepearce@phcn.vic.gov.au

**Please list the external (Non-Monash) investigators involved.**

*You cannot search the Monash directory for non-Monash investigators or share the project with them as access to ERM is restricted to Monash staff and students. You need to enter the details below manually.*

| Title | First Name | Surname |
|---|---|---|
| Ms | Stacey | Aslangul |

Organisation: St Kilda Gatehouse

Email: stacey@stkildagatehouse.org.au

**Please list the external (Non-Monash) investigators involved.**

*You cannot search the Monash directory for non-Monash investigators or share the project with them as access to ERM is restricted to Monash staff and students. You need to enter the details below manually.*

| Title | First Name | Surname |
|---|---|---|
| Mr | Jim | Panakos |

Organisation: St Kilda Gatehouse

Email: jim@stkildagatehouse.org.au

**Please list the external (Non-Monash) investigators involved.**

*You cannot search the Monash directory for non-Monash investigators or share the project with them as access to ERM is restricted to Monash staff and students. You need to enter the details below manually.*

| Title | First Name | Surname |
|---|---|---|
| Ms | Fiona | Cowan |

Organisation: St Kilda Gatehouse

Email: fiona@stkildagatehouse.org.au

**Please list the external (Non-Monash) investigators involved.**

*You cannot search the Monash directory for non-Monash investigators or share the project with them as access to ERM is restricted to Monash staff and students. You need to enter the details below manually.*

| Title | First Name | Surname |
|---|---|---|
| Ms | Lucy | Maio |

Organisation: St Kilda Gatehouse

Email: lucy@stkildagatehouse.org.au

**Please list the external (Non-Monash) investigators involved.**

*You cannot search the Monash directory for non-Monash investigators or share the project with them as access to ERM is restricted to Monash staff and students. You need to enter the details below manually.*

| Title | First Name | Surname |
|---|---|---|
| Ms | Megan | Robinson |

Organisation: St Kilda Gatehouse

Email: megan@stkildagatehouse.org.au

**Please list the external (Non-Monash) investigators involved.**

*You cannot search the Monash directory for non-Monash investigators or share the project with them as access to ERM is restricted to Monash staff and students. You need to enter the details below manually.*

| Title | First Name | Surname |
|---|---|---|
| Ms | Claire | Fraser |

Organisation: Able Australia

Email: claire.fraser@ableaustralia.org.au

**Please list the external (Non-Monash) investigators involved.**

*You cannot search the Monash directory for non-Monash investigators or share the project with them as access to ERM is restricted to Monash staff and students. You need to enter the details below manually.*

| Title | First Name | Surname |
|---|---|---|
| Ms | Golsum | Ramezani |

Organisation: enliven

Email: golsum2020@gmail.com

**Please list the external (Non-Monash) investigators involved.**

*You cannot search the Monash directory for non-Monash investigators or share the project with them as access to ERM is restricted to Monash staff and students. You need to enter the details below manually.*

| Title | First Name | Surname |
|---|---|---|
| Ms | Thanuja | Herath |

Organisation: Wellsprings for women

Email: thanuja@wellspringsforwomen.com

**Please list the external (Non-Monash) investigators involved.**

*You cannot search the Monash directory for non-Monash investigators or share the project with them as access to ERM is restricted to Monash staff and students. You need to enter the details below manually.*

| Title | First Name | Surname |
|---|---|---|
| Ms | Chithrika | Senanayake |

Organisation: Wellsprings for women

Email: chithrika45@gmail.com

**Please list the external (Non-Monash) investigators involved.**

*You cannot search the Monash directory for non-Monash investigators or share the project with them as access to ERM is restricted to Monash staff and students. You need to enter the details below manually.*

| Title | First Name | Surname |
|---|---|---|
| Ms | Silva | Nazaretian |

Organisation: Enliven

Email: silva@enliven.org.au

**A5 Briefly outline the experience and qualifications of the research team that are necessary for the conduct of this research.**

*Maximum of 1500 characters including spaces.*

CI Haines–Health services researcher, provided leadership to projects of >$50 million value. Quantitative researcher. CI Russell–refugee and asylum seeker group Lead, GP, skills in mixed methods. CI Hill- aged care worker group Lead, physiotherapist, gerontology researcher. CI Williams– ATSI group Lead, clinician researcher co-located at Peninsula Health, supported Peninsula Health to establish ATSI Health service. CI Callaway– people living with disability and carers group Lead, Occupational Therapist. CI Hlavac– linguistically diverse group Lead, certified interpreter/educator, extensive experience in development of multi-modal information to speakers of languages other than English. CI Willoughby– Lead of deaf and hard of hearing subgroup. Applied linguist, focuses on how mainstream institutions communicate with consumers from non-English speaking backgrounds. CI Long– Postdoctoral researcher with significant experience in mixed-methods research and project management. CI Loads- Media communications. CI Robins- Project Manager. Postdoctoral researcher. CI Lennon– Lead of street-based sex workers subgroup. Previous research with this sub-group.CI Pearce - connections with local Aboriginal community and Indigenous himself. Aboriginal Liaison Officer at Peninsula Health. CI Aslangul- Gatehouse CEO. CI's Panakos, Cowan, Maio, Robinson- Gatehouse Drop In Support workers. CI Beaver- project officer at Deaf Victoria. CI Nazaretian-Enliven equity access and diversity manager.

## Background and Aims

**A6 In plain language, provide a succinct description of the background and the potential significance of the research project.**

- *Maximum of 2000 characters, including spaces.*
- *Do not include citations and explain all abbreviations and avoid using complex technical terms.*

Effective health mass media campaign that deliver well-designed and relevant messages to all communities in Australia are needed to help manage the COVID-19 and other future pandemics. It is unknown however, how these campaigns should be best constructed and delivered to the general public and vulnerable populations within it. In stage 1 of this project we have identified COVID-19 information gathering sources and needs of vulnerable populations and the general public. Based on these findings we have now developed tailored communication approaches to address common needs. We plan to test and compare their effectiveness in the hope of identifying the more effective communication strategies. The specific strategies that we will be testing in this research include;
1. use of animation versus 'talking head' visual stimulus approaches
2. Use of a general practitioner to deliver the COVID-19 relevant information versus a community representative (for our vulnerable subgroups) or versus a combination of politicians and chief medical officers (for our general community sample)
3. Focusing the material on information provision versus focusing the material on conversation training
These interventions were selected as our project co-production team representing our vulnerable subgroup populations identified the need to test conversation training and use of animation as high priority questions to be addressed. Our stage 1 survey also identified general practitioners as being the most highly trusted source of COVID-19 information.
4. Understanding if a person is more likely to change their behaviour after watching an education video about COVID-19 if they felt they were strongly represented in the video.

**A6(i) Please clarify how this project incorporates the core values of research with Aboriginal and Torres Strait Islander Peoples.**

*Please refer to the blue 'Info' icon on the right-hand side for links to the guidelines.*

Spirit and integrity: We are committed to conducting our research combining all the other five core values (cultural continuity, equity, reciprocity, respect, and responsibility), to demonstrate spiritual integrity. Our research aims to identify health information needs of ATSI people in a way that is honest and recognises the principles of research conduct.
Cultural Continuity & Respect: By recruiting CI's established in working with ATSI people both clinically and in research, we can ensure cultural preservation, and appropriate engagement with our ATSI participants, demonstrating cultural continuity. This process will also aid in ensuring we have developed trust, openness and engagement between potential participants and the research being conducted also demonstrating respect.
Equity & Reciprocity : Our research aim is to enhance our understanding on the health messaging needs of ATSI and other subgroup populations within Australia, and to adapt current health messaging to better suit the needs of these subgroups. We hope that the findings of our research will have a great health benefit to the existing and future ATSI community.
Responsibility: It is not expected that this research will cause any harm to any participant including ATSI participants. All participants will be required to provide informed consent before participating and all data collected will be completely confidential and unable to be identified. Any results produced from this research will be provided to participants only if they choose to receive results from this research.

A6(ii) **Please attach evidence of support from the Aboriginal and Torres Strait Islander communities involved in this research.**

*To avoid delays in approving the project, it's important that evidence of support is included.*

Documents

| Type | Document Name | File Name | Version Date | Version | Size |
|---|---|---|---|---|---|
| Supporting Documentation | MRFF CSADO Grant RRCSA000014_Partner Letter_Peninsula Health | MRFF CSADO Grant RRCSA000014_Partner Letter_Peninsula Health.pdf | 23/09/2021 | V1 | 169.7 KB |

**A7 Clearly state the aims and/or hypotheses of the research project.**

- *Maximum of 2000 characters, including spaces.*
- *Do not include citations and explain all abbreviations and avoid using complex technical terms.*

Question 1: Does providing COVID-19 information using animation increase intention to follow COVID safe behaviours and encourage others to do likewise compared to a non-animation ('talking head') control?
Question 2: Does providing COVID-19 information using a general practitioner to present the information increase intention to follow COVID safe behaviours and encourage others to do likewise compared to a community representative presenting this same information?
Question 3: Does providing COVID-19 information using a conversation training approach increase intention to follow COVID safe behaviours and encourage others to do likewise compared to an information provision approach?

These questions will be examined using the entire sample and with subgroup analyses separating each vulnerable population from the general community sample.
A further subgroup analyses will be undertaken to examine the moderating effect of being a member of a COVID risk latent class (as identified in stage 1 of our research). Membership of COVID risk latent classes is determined by responses to items within the demographic questions examining self reported likelihood in participating in COVID-19 safe behaviours.

## Research Scope

**A8 This research involves:**

*Tick all that apply. Please refer to the blue 'Info' icon for information about existing data.*

- [x] Prospective recruitment or observation of human participants
- [ ] Use of existing data or use of existing biospecimens

**A8(i) The project also involves:**

*Tick all that apply.*

- ☐ Clinical Trial*
- ☐ Genetic testing or analysis of genetic material
- ☐ Administration of ionising radiation - e.g. X-ray, DEXA scan

** A clinical trial is defined by the World Health Organisation (WHO) as 'any study that prospectively assigns human participants or groups of humans to one or more health-related interventions to evaluate the effects on health outcomes'.*

## Benefits and Risks

**A9 Please outline the benefits to participants and to the community as a result of this research being conducted.**

We hope that our research is able to identify the more effective COVID-19 health messaging approaches for specific subgroup populations within our Australian community. This will then inform future government health messaging strategies and ensure that they are as effective as possible for these specific subgroups as well as the general community as a whole. Subgroups include: Refugee and asylum seekers, aged care workers, ATSI, people living with disability and their carers, deaf and hearing impaired people, and street based sex workers. We hope that this research may benefit participants directly, by providing further education around COVID-19, keeping themselves, families and communities safe from COVID-19, and provide insight into how to best produce other health messaging for these specific groups in the future.

## Risks

**A11 Outline any potential risks, in either the short or long term, of participation in this project.**

*E.g. physical, psychological, social, economic or legal risks to participants greater than inconvenience or discomfort.*

Participation in completing this survey is low risk, and no adverse events are excepted including any psychological distress throughout its completion. In the case that psychological/emotional discomfort is experienced by a participant answering survey questions, they are able to stop the survey immediately and a) not return to complete it, automatically withdrawing themselves from the research, or b) close the window and return to the survey up to 1 week later to complete it. In the event that a participant feels psychological or emotional discomfort from answering questions related to COVID-19, they are able to contact Lifeline or their local support person via the details found in each subgroup explanatory statement. The contact details of the chief investigator and executive officer are also provided on the explanatory statement if participants prefer to contact the research team directly.

**A12 Are all these risks outlined on the Explanatory Statement and, where relevant, on the Consent Form?**

- ◉ Yes
- ○ No

**A13 Is an appropriate list of counselling services included in the Explanatory Statement?**

- ◉ Yes
- ○ No

**A14 Outline the arrangements planned to minimise the risks involved in these procedures.**

Recruiting CI's that have established relationships with organisations involved with our target population will assist in developing trust and rapport when recruiting participants and discussing this research with them.

## Risk Management

**A15 What will you do in cases where serious events or emergencies occur as a result of participation in this project and what facilities are available to deal with such incidents?**

In the case that psychological/emotional discomfort is experienced by a participant answering survey questions, they are able to stop the survey immediately and a) not return to complete it, automatically withdrawing themselves from the research, or b) close the window and return to the survey up to 1 week later to complete it. In the event that a participant feels psychological or emotional discomfort from answering questions related to COVID-19, they are able to contact the research team via the details outlined in the explanatory statement. Appropriate details for counselling services can then be provided to the participant as required.
If a serious event or emergency occurs, members of the research team/partner organisation will intervene where applicable including calling 000 if necessary. However these kinds of emergencies are not anticipated.

**A17 Is this research project likely to reveal information relating to child sexual abuse that must be disclosed to police?**

- ○ Yes
- ◉ No

## Project Details

**A18 Is this project related to other Monash University human ethics applications?**

- ◉ Yes
- ○ No

*Please provide the MUHREC approval number(s) and explain the nature of the relationship between the current application and the previous application(s).*

Application MUHREC 27804 is Stage 1 of this project, and provided the research evidence to inform this next phase (Stage 3) of the project .

**A18(i) Is this project replacing the above application because it expired?**

- ○ Yes
- ◉ No

**A19 Will this project be submitted to other Human Research Ethics Committees (HRECs)?**

*If the research has already been approved by another HREC, please stop completing this form and register the project with MUHREC using the "Other HREC" registration form.*

- ( ) Yes
- (•) No

## Type of Research

**A20 This research is a:**

- [x] Staff project
- [ ] Student project
- [ ] Unit project

**A21 Type of Research - 2**

- [ ] Action Research
- [ ] Epidemiological
- [x] Public Health and Safety
- [ ] Case study
- [ ] Oral history / Biographical
- [ ] Clinical Trial/use of drug or device
- [ ] Clinical Research
- [ ] Medical Research
- [x] Quantitative
- [ ] Qualitative
- [ ] Social Science
- [ ] Other

## Other Organisations

**A22 Does your research involve recruiting participants or obtaining data from organisations other than Monash University?**

- (•) Yes
- ( ) No

*Please provide details in the table below. The contact person is someone who can provide permission for the research to be conducted at each organisation. This cannot be anyone listed on the research team in section A as it would be considered a conflict of interest.*

| Organisation | Contact name | Position |
| --- | --- | --- |
| Able Australia | Meredith Prain- meredith.prain@ableaustralia.org.au | Deaf/blindness consultant |

----------------------------------------------------------------------------------

*To add another organisation, click the "Add another" button.*

*Please provide details in the table below. The contact person is someone who can provide permission for the research to be conducted at each organisation. This cannot be anyone listed on the research team in section A as it would be considered a conflict of interest.*

| Organisation | Contact name | Position |
|---|---|---|
| Wallara | Phil Hayes-Brown-phayesbrown@wallara.com.au<br>Thera Storie-tstorie@wallara.com.au | CEO<br>General Manager |

----------------------------------------------------------------------------------

*To add another organisation, click the "Add another" button.*

*Please provide details in the table below. The contact person is someone who can provide permission for the research to be conducted at each organisation. This cannot be anyone listed on the research team in section A as it would be considered a conflict of interest.*

| Organisation | Contact name | Position |
|---|---|---|
| Yooralla | Leanne Turner-leanne.turner@yooralla.com.au<br><br>Paul Ireland-paul.Ireland@yooralla.com.au | Executive Director Residential and Respite support services.<br>Director, policy, compliance and research |

----------------------------------------------------------------------------------

*To add another organisation, click the "Add another" button.*

*Please provide details in the table below. The contact person is someone who can provide permission for the research to be conducted at each organisation. This cannot be anyone listed on the research team in section A as it would be considered a conflict of interest.*

| Organisation | Contact name | Position |
|---|---|---|
| Donwood Community Ages Care Services | Natasha Wilkinson-nwilkinson@donwood.com.au | CEO |

----------------------------------------------------------------------------------

*To add another organisation, click the "Add another" button.*

*Please provide details in the table below. The contact person is someone who can provide permission for the research to be conducted at each organisation. This cannot be anyone listed on the research team in section A as it would be considered a conflict of interest.*

| Organisation | Contact name | Position |
|---|---|---|
| enliven | Rob Macindoe-rob@enliven.org.au | Executive Director |

----------------------------------------------------------------------------------

*To add another organisation, click the "Add another" button.*

*Please provide details in the table below. The contact person is someone who can provide permission for the research to be conducted at each organisation. This cannot be anyone listed on the research team in section A as it would be considered a conflict of interest.*

| Organisation | Contact name | Position |
|---|---|---|
| Peninsula Health | Iain Edwards-iedwards@phcn.vic.gov.au<br>Leanne Murray-LMurray@phcn.vic.gov.au<br>Helen Bnads-HBnads@phcn.vic.gov.au | Operations Director-Community Health<br><br>Koolin Balit Co-ordinator |

----------------------------------------------------------------------------------

*To add another organisation, click the "Add another" button.*

*Please provide details in the table below. The contact person is someone who can provide permission for the research to be conducted at each organisation. This cannot be anyone listed on the research team in section A as it would be considered a conflict of interest.*

| Organisation | Contact name | Position |
|---|---|---|
| St Kilda Gatehouse | Dianne Azoor Hughes- dianne@stkildagatehouse.org.au | Chair of the board |

------------------------------------------------------------------------------------

*To add another organisation, click the "Add another" button.*

*Please provide details in the table below. The contact person is someone who can provide permission for the research to be conducted at each organisation. This cannot be anyone listed on the research team in section A as it would be considered a conflict of interest.*

| Organisation | Contact name | Position |
|---|---|---|
| Boroondara Aged Services Society (BASScare) | Sue Bowditch- resmgr@basscare.org.au | Residential Manager |

------------------------------------------------------------------------------------

*To add another organisation, click the "Add another" button.*

*Please provide details in the table below. The contact person is someone who can provide permission for the research to be conducted at each organisation. This cannot be anyone listed on the research team in section A as it would be considered a conflict of interest.*

| Organisation | Contact name | Position |
|---|---|---|
| Samarinda Ashburton Aged Services | Angela Rako- angelar@saas.org.au | Quality and learning coordinator |

------------------------------------------------------------------------------------

*To add another organisation, click the "Add another" button.*

*Please provide details in the table below. The contact person is someone who can provide permission for the research to be conducted at each organisation. This cannot be anyone listed on the research team in section A as it would be considered a conflict of interest.*

| Organisation | Contact name | Position |
|---|---|---|
| Ringwood Area Lions Aged Care (RALAC) | Chris Reside- ceo@ralac.org.au | CEO |

------------------------------------------------------------------------------------

*To add another organisation, click the "Add another" button.*

*Please provide details in the table below. The contact person is someone who can provide permission for the research to be conducted at each organisation. This cannot be anyone listed on the research team in section A as it would be considered a conflict of interest.*

| Organisation | Contact name | Position |
|---|---|---|
| Eva Tilley Memorial Home | Kaye Brown- QualityManager@evatilley.com.au | Quality Manager |

------------------------------------------------------------------------------------

*To add another organisation, click the "Add another" button.*

## Financial Details

**A23 Do any of the investigators have a personal or financial interest in a) the outcome of this research, b) any of the organisations involved with the research, or c) any of the organisations funding this project?**

- ○ Yes
- ◉ No

**A24 Has external funding been obtained for this project?**

- ◉ Yes
- ○ No

*Provide the name of the funding body or agency and PURE ID number.*

Medical Research Future Fund (MRFF).
Pure ID: 325803478

**A24(i) Has the funding source and details been fully disclosed to participants in the Explanatory Statement and will this be disclosed in any subsequent research output?**

- ◉ Yes
- ○ No

## Participant Groups

**B1 Describe who the participants in each group are and where they will be recruited from.**

*For projects involving participants in other countries, please ensure that you are aware of any requirements for review by local ethics committees and obtain the appropriate permissions prior to commencing recruitment / data collection.*

| Participant group | Recruited from | No. of participants |
|---|---|---|
| People Living with a disability and their carers | Able Australia<br>Wallara<br>Yooralla | 40 |

---------------------------------------------------------------------------------

*To add another group, click the "Add Another" button below.*

**B1 Describe who the participants in each group are and where they will be recruited from.**

*For projects involving participants in other countries, please ensure that you are aware of any requirements for review by local ethics committees and obtain the appropriate permissions prior to commencing recruitment / data collection.*

| Participant group | Recruited from | No. of participants |
|---|---|---|
| Aged Care Workers | Donwood Community Aged Care Services<br>Boroondara Aged Services Society (BASScare)<br>Samarinda Ashburton Aged Services<br>Ringwood Area Lions Aged Care (RALAC)<br>Eva Tilley Memorial Home | 40 |

---------------------------------------------------------------------------------

*To add another group, click the "Add Another" button below.*

**B1 Describe who the participants in each group are and where they will be recruited from.**

*For projects involving participants in other countries, please ensure that you are aware of any requirements for review by local ethics committees and obtain the appropriate permissions prior to commencing recruitment / data collection.*

| Participant group | Recruited from | No. of participants |
|---|---|---|
| Refugee and asylum seekers | enliven | 40 |

---

*To add another group, click the "Add Another" button below.*

**B1 Describe who the participants in each group are and where they will be recruited from.**

*For projects involving participants in other countries, please ensure that you are aware of any requirements for review by local ethics committees and obtain the appropriate permissions prior to commencing recruitment / data collection.*

| Participant group | Recruited from | No. of participants |
|---|---|---|
| Deaf, hard of hearing people | Expression Australia | 40 |

---

*To add another group, click the "Add Another" button below.*

**B1 Describe who the participants in each group are and where they will be recruited from.**

*For projects involving participants in other countries, please ensure that you are aware of any requirements for review by local ethics committees and obtain the appropriate permissions prior to commencing recruitment / data collection.*

| Participant group | Recruited from | No. of participants |
|---|---|---|
| Aboriginal and Torres Strait Islander people | Local Kulin Land Gathering/Meeting Places: Narim Marr Djambana Gathering Place (Frankston) and Willum Warrain Meeting Place (Hastings). | 40 |

---

*To add another group, click the "Add Another" button below.*

**B1 Describe who the participants in each group are and where they will be recruited from.**

*For projects involving participants in other countries, please ensure that you are aware of any requirements for review by local ethics committees and obtain the appropriate permissions prior to commencing recruitment / data collection.*

| Participant group | Recruited from | No. of participants |
|---|---|---|
| Street based sex workers | St Kilda Gatehouse | 40 |

---

*To add another group, click the "Add Another" button below.*

**B1 Describe who the participants in each group are and where they will be recruited from.**

*For projects involving participants in other countries, please ensure that you are aware of any requirements for review by local ethics committees and obtain the appropriate permissions prior to commencing recruitment / data collection.*

| Participant group | Recruited from | No. of participants |
|---|---|---|
| General community | Survey link will be advertised using Monash University facebook and twitter platforms.<br>Participants in Monash University COVID-19 survey conducted in 2020 who indicated their consent to be contacted for further research will also be invited to participate. | Minimum of 300. Given the social media advertising approach that we will use it is highly likely that more than 300 will be recruited prior to the paid advertising run being completed. |

---

*To add another group, click the "Add Another" button below.*

**B2 Are any participants under 18 years of age?**

*A Working with Children Check is now required whether contact with children is supervised or not. Click the blue 'Info' icon on the right for further details.*

- ( ) Yes
- (•) No

## Participant Details

**B5 Is there a pre-existing (unequal) relationship between anyone involved in recruiting and/or collecting data and anyone from any of the participant groups?**

*E.g. teachers/students, health care providers/patients.*

- (•) Yes
- ( ) No

*Describe the nature of the relationship, and explain what special precautions will preserve the rights of such people to decline to participate or to withdraw from participation once the research has begun.*

The relationship between our partner organisations and potential participants is best described as a community relationship. Our partner organisations provide support, access to various services and a 'safe place' for people of different population groups to come and ask for help and support. Donwood Community Aged Care Services is the only exception where this partner organisation is the employer of potential participants for this subgroup population. Researchers recruiting at each partner organisation will inform potential participants of the research via: 1. an information flyer, or 2. word of mouth, or 3. email, or 4. social media. Potential participants will have to nominate themselves if they wish to participate (across all subgroups). This will preserve the rights of all people to decline to participate, or withdraw from the research after its commencement.

**B6 Does this research involve people in countries other than Australia?**

- ( ) Yes
- (•) No

**B7 Do any of the participant groups listed in B1 have any cultural sensitivities that need to be considered in the design of this research?**

- (•) Yes
- ( ) No

*Please outline the arrangements you have in place for managing these cultural needs.*

By enlisting the assistance of partner organisations such as Peninsula Health (and their associated contact people) for the ATSI community, Enliven for refugee and asylum seekers, and Able Australia for people living with a disability, we can ensure our research methodologies demonstrate research merit and integrity, justice, beneficence and respect. Consent for example will only be provided by those with the capacity to do so, from either the participant themselves, or from a legal guardian.

**B8 Do you have any criteria for exclusion from any of the participant groups listed in B1?**

- (•) Yes
- ( ) No

*Please list the relevant groups and describe and justify this exclusion.*

| Group (as listed in B1) | Exclusion criteria |
| --- | --- |
| People living with a disability and their carers | If the person wanting to participate is less than 18 years of age they will be excluded from participating as we are only looking to recruit adults.<br>If the person wanting to participate currently lives outside of Australia they will also be excluded as for the purpose of this research, we are only looking at those who reside in Australia. |

---

*To add another group, click the "Add Another" button below.*

*Please list the relevant groups and describe and justify this exclusion.*

| Group (as listed in B1) | Exclusion criteria |
| --- | --- |
| Aged Care Workers | If the person wanting to participate is less than 18 years of age they will be excluded from participating as we are only looking to recruit adults.<br>If the person wanting to participate currently lives outside of Australia they will also be excluded as for the purpose of this research, we are only looking at those who reside in Australia. |

---

*To add another group, click the "Add Another" button below.*

*Please list the relevant groups and describe and justify this exclusion.*

| Group (as listed in B1) | Exclusion criteria |
| --- | --- |
| Refugee & asylum seekers | If the person wanting to participate is less than 18 years of age they will be excluded from participating as we are only looking to recruit adults.<br>If the person wanting to participate currently lives outside of Australia they will also be excluded as for the purpose of this research, we are only looking at those who reside in Australia.<br>If the participant is unable to speak/read English, Dari or Sinhalese they will also be excluded as the English survey will only be translated to Dari and Sinhalese languages outside of English. |

---

*To add another group, click the "Add Another" button below.*

*Please list the relevant groups and describe and justify this exclusion.*

| Group (as listed in B1) | Exclusion criteria |
| --- | --- |
| Deaf, hard of hearing people | If the person wanting to participate is less than 18 years of age they will be excluded from participating as we are only looking to recruit adults.<br>If the person wanting to participate currently lives outside of Australia they will also be excluded as for the purpose of this research, we are only looking at those who reside in Australia. |

---

*To add another group, click the "Add Another" button below.*

*Please list the relevant groups and describe and justify this exclusion.*

| Group (as listed in B1) | Exclusion criteria |
| --- | --- |
| Aboriginal & Torres Strait Islander people | If the person wanting to participate is less than 18 years of age they will be excluded from participating as we are only looking to recruit adults.<br>If the person wanting to participate currently lives outside of Australia they will also be excluded as for the purpose of this research, we are only looking at those who reside in Australia. |

---

*To add another group, click the "Add Another" button below.*

*Please list the relevant groups and describe and justify this exclusion.*

| Group (as listed in B1) | Exclusion criteria |
| --- | --- |
| Street-based sex workers | If the person wanting to participate is less than 18 years of age they will be excluded from participating as we are only looking to recruit adults.<br>If the person wanting to participate currently lives outside of Australia they will also be excluded as for the purpose of this research, we are only looking at those who reside in Australia. |

---------------------------------------------------------------------------------------------------------

*To add another group, click the "Add Another" button below.*

*Please list the relevant groups and describe and justify this exclusion.*

| Group (as listed in B1) | Exclusion criteria |
| --- | --- |
| General community | If the person wanting to participate is less than 18 years of age they will be excluded from participating as we are only looking to recruit adults.<br>If the person wanting to participate currently lives outside of Australia they will also be excluded as for the purpose of this research, we are only looking at those who reside in Australia. |

---------------------------------------------------------------------------------------------------------

*To add another group, click the "Add Another" button below.*

## Recruitment Methods

### B9 Indicate which method(s) you will use to recruit participants for this research:

- ☑ Email
- ☐ Personal contacts
- ☐ Advertisement
- ☑ Participants from previous study
- ☐ Participants will be observed without their knowledge and will not be actively recruited
- ☐ Mail out
- ☐ Snowballing (participants passing the research invite to others)
- ☐ Telephone
- ☑ Participants approached in person
- ☑ Other (e.g. social media, using research panel companies)

For email recruitment, please note that you cannot recruit students enrolled at Monash University Malaysia campus via their Monash student email accounts or via emailing them in Moodle.

*For each method selected above, please clarify how you will recruit the participants.*

Participants will be recruited from partner organisations listed previously and in more detail below. A specific script will be used initially to gain expression of interest (via email, in person or via the partner organisation social media pages), explaining the basic details about the research project and what is required (see attached). The easy to read explanatory statement will be translated into the required languages for Refugee and asylum seeker participants and also into AUSLAN for the deaf and hard-of-hearing group. General community participants will be recruited via advertising on Monash University Facebook and Twitter platforms.

*Participants from previous study: please clarify how participants have consented to being contacted for future research (e.g. this project) and upload copies of previous consent forms below.*

*If you are recruiting participants from a MUHREC approved participant pool, indicate the name of that pool and contact their administrator and provide the ethics approval number for that pool below.*

Participants who participated in Stage 1 of this project had the option of providing their contact details to be contacted for future research if they were interested. The questions included were:
"Are you happy for us to contact you for future stages of this research related to COVID-19"? Participants had the option of responding "yes" or "no". If they selected "yes"' they were then asked to provide their contact details to be contacted for future research.

*Please upload copies of the documents or information you will use for recruitment or advertisement (e.g email invite, flyers, social media posts, emails to personal contacts, phone script etc...).*

*Please refer to the Monash Advertisement template and modify to suit your project.*

Documents

| Type | Document Name | File Name | Version Date | Version | Size |
|---|---|---|---|---|---|
| Supporting Documentation | Previous Participants Recruitment Email_V1_21102021 | Previous Participants Recruitment Email_V1_21102021.docx | 24/02/2022 | V1 | 15.2 KB |
| Supporting Documentation | STAGE 1 RESULTS SUMMARY for recruitment | STAGE 1 RESULTS SUMMARY for recruitment.pdf | 24/02/2022 | V1 | 1.0 MB |

## Inviting Participants

### B10 Indicate how you will obtain the contact details of potential participants:

- [x] From the participants themselves
- [ ] From a public domain source
- [ ] From a private or third party source
- [x] Other

*Other: Please clarify how you will obtain these contact details.*

Contact details have been obtained from previous survey responses where the participant indicated that they would like to be contacted for "future research related to COVID-19". The participants then provided their contact details.

**B11 For each group listed in B1, please explain who will send the invitation to potential participants, how they will be invited and when they will be given the Explanatory Statement.**

| Participant group | Invite details |
|---|---|
| People living with disability and their carers | Able Australia- Recruitment will take place via Able Australia's service managers at key SDA and lifestyle program sites providing a one-page (plain English/easy read) study overview/information sheet and associated survey link for participants to follow.<br><br>Wallara- Recruitment to take place through Wallara service managers at key supported employment and lifestyle program sites providing a one-page plain English study information sheet and associated survey link for participants to follow. Wallara will provide support to engage people and facilitate completion of surveys where required.<br>Yooralla- Yooralla’s marketing and communications team will be able to promote the study to recruit people with disability using the plain English study information sheet developed by the research team. Yooralla will also provide links to the survey for participants to then complete.<br><br>At each of these sites, a script will be used to assist in introducing the research to potential participants which will then lead to further information in the 'Easy English' explanatory statement at the beginning of the survey. The more detailed explanatory statement will also be made available and be imbedded within the 'Easy English' statement at this time before consent is provided.<br><br>An email invitation will also be sent to participants of a previous Monash University COVID-19 survey (ethics application 30698) who consented to be contacted for further research.<br><br>Although we are recruiting Aboriginal and Torres Strait Islander people as their own subgroup, it is still possible that they also might fit into the subgroup population of people living with disability and their carers. In this instance we will be providing them the survey specific to people living with disability and their carers. |

**B11 For each group listed in B1, please explain who will send the invitation to potential participants, how they will be invited and when they will be given the Explanatory Statement.**

| Participant group | Invite details |
|---|---|
| Aged care workers | Donwood Community Aged Care Services- will use their contacts/networks within the organisations to recruit potential participants and provide the link to the survey to then be completed by participants. This will be done via word of mouth, in meetings and emails.<br>A short paragraph will be used to assist in introducing the research to potential participants which will then lead to further information in the 'Easy English' explanatory statement at the beginning of the survey. The more detailed explanatory statement will also be made available and be imbedded within the 'Easy English' statement at this time before consent is provided.<br>An email invitation will also be sent to participants of a previous Monash University COVID-19 survey (ethics application 30698) who consented to be contacted for further research.<br><br>Although we are recruiting Aboriginal and Torres Strait Islander people as their own subgroup, it is still possible that they also might fit into the subgroup population of aged care workers. In this instance we will be providing them the survey specific to aged care workers. |

**B11 For each group listed in B1, please explain who will send the invitation to potential participants, how they will be invited and when they will be given the Explanatory Statement.**

| Participant group | Invite details |
|---|---|
| Refugee and asylum seekers | enliven- will use their contacts/networks within the organisations to recruit potential participants and provide the link to the survey to then be completed by participants. This will include recruitment of a Bi-Cultural Worker to assist in networking with relevant community groups for recruitment purposes.<br>A script will be used to assist in introducing the research to potential participants which will then lead to further information in the 'Easy English' explanatory statement at the beginning of the survey. The more detailed explanatory statement will also be made available and be imbedded within the 'Easy English' statement at this time before consent is provided.<br>Any written material provided to potential participants will be available to be translated to a preferred language (Dari or Sinhalese) as required.<br>An email invitation will also be sent to participants of a previous Monash University COVID-19 survey (ethics application 30698) who consented to be contacted for further research. |

**B11 For each group listed in B1, please explain who will send the invitation to potential participants, how they will be invited and when they will be given the Explanatory Statement.**

| Participant group | Invite details |
|---|---|
| Aboriginal and Torres Strait Islander people | Peninsula Health-Participants will be recruited via the site's Aboriginal liaison officer using the plain English script and explanatory statement sheet provided by the research team and verbal discussions.<br>A link to the 'Easy English' explanatory statement will be provided which will then link them to providing consent and completing the survey. The more detailed explanatory statement will also be made available and be imbedded within the 'Easy English' statement at this time before consent is provided.<br>Where required iPads will be issues to Peninsula Health to assist in the completion of the online surveys.<br>An email invitation will also be sent to participants of a previous Monash University COVID-19 survey (ethics application 30698) who consented to be contacted for further research. |

**B11 For each group listed in B1, please explain who will send the invitation to potential participants, how they will be invited and when they will be given the Explanatory Statement.**

| Participant group | Invite details |
|---|---|
| Deaf, hard of hearing people | Expression Australia- Recruitment to take place through Expression Australia's Facebook page using a one-page (plain English/easy read) study overview/information sheet provided by the research team. AUSLAN interpretation in a video link will also be provided via the research assistant in conjunction with Expression Australia to assist with recruitment also.<br>A script (that will be used in the Facebook post) will be used to assist in introducing the research to potential participants which will then lead to further information in the 'Easy English' explanatory statement at the beginning of the survey as potential participants follow the link.<br>The more detailed explanatory statement will also be made available and be imbedded within the 'Easy English' statement at this time before consent is provided.<br>Although we are recruiting Aboriginal and Torres Strait Islander people as their own subgroup, it is still possible that they also might fit into the subgroup population of deaf, hard of hearing people. In this instance we will be providing them the survey specific to deaf, hard of hearing people.<br>An email invitation will also be sent to participants of a previous Monash University COVID-19 survey (ethics application 30698) who consented to be contacted for further research. |

**B11 For each group listed in B1, please explain who will send the invitation to potential participants, how they will be invited and when they will be given the Explanatory Statement.**

| Participant group | Invite details |
| --- | --- |
| Street-based sex workers | St Kilda Gatehouse-will use their contacts/networks within the organisations to recruit potential participants and provide the link to the survey to then be completed by participants. This will be done mostly via word of mouth. The organisation (St Kilda Gatehouse) will provide support to participants to complete the survey where required. iPads will be issued to St Kilda Gatehouse to assist in completing the Online survey on site.<br><br>A script can be used to assist in introducing the research to potential participants which will then lead to further information in the 'Easy English' explanatory statement at the beginning of the survey. The more detailed explanatory statement will also be made available and be imbedded within the 'Easy English' statement at this time before consent is provided. If consent at this point is provided, it will then lead the participant to the survey questions.<br>Although we are recruiting Aboriginal and Torres Strait Islander people as their own subgroup, it is still possible that they also might fit into the subgroup population of street-based sex workers. In this instance we will be providing them the survey specific to street-based sex workers.<br>An email invitation will also be sent to participants of a previous Monash University COVID-19 survey (ethics application 30698) who consented to be contacted for further research and provided adequate contact details. |

**B11 For each group listed in B1, please explain who will send the invitation to potential participants, how they will be invited and when they will be given the Explanatory Statement.**

| Participant group | Invite details |
| --- | --- |
| General community | An invitation to participate in the survey will be advertised using Monash University Facebook and Twitter platforms. A link to the survey will be provided in the advertisement. This will allow interested parties to read the explanatory statement in the survey and to provide consent before continuing.<br>This is an example of the type of message that will be sent: Our research team are interested to find out whether vulnerable populations respond differently to COVID-19 messaging compared to the general community. Your survey responses will help vulnerable people by helping us develop better targeted messaging. Survey info and link: (provide survey link here).<br>An email invitation will also be sent to participants of a previous Monash University COVID-19 survey (ethics application 30698) who consented to be contacted for further research. |

## Reimbursement of Participants

**B12 Will you be offering payment or any other incentives to any of the participants?**

*For more information, refer to the NHMRC advice on payment of participants in research.*

- (•) Yes
- ( ) No

*Please explain how much, what form the incentive will take and justify why this will not be an inducement.*

*Maximum 2000 characters, including spaces.*

The survey is expected to take approximately 30-60mins, as various subgroup populations may have specific challenges such as English as a second language or low literacy levels, which may make completing the survey a challenge. Thus, to thank participants for their effort and time, on completion of the vulnerable subgroup survey the first 40 participants will be provided with or sent (email or post) a $50 gift card. Participants from the general community group will go into a draw to win one of 20 gift voucher prizes to the value of $50 each.

## Explanatory Statement

**B13 Will you use a written Explanatory Statement to inform the participants about this project?**

*Please refer to the Monash Explanatory Statement template and modify to suit your project.*

◉ Yes
○ No

*Please upload a copy of the Explanatory Statement(s). If you have multiple documents for different groups of participants, please clearly label each Explanatory Statement for ease of reference. To upload more than one document, click the "Upload Document".*

Documents

| Type | Document Name | File Name | Version Date | Version | Size |
|---|---|---|---|---|---|
| Explanatory Statement | Easy to read Explanatory statement Aged Care workers_V2 | Easy to read Explanatory statement Aged Care workers_V2.docx | 31/03/2022 | V2 | 116.7 KB |
| Explanatory Statement | Long EXPLANATORY STATEMENT Aged care workers_V2 | Long EXPLANATORY STATEMENT Aged care workers_V2.docx | 31/03/2022 | V2 | 118.6 KB |
| Explanatory Statement | STAGE 3 Easy to read Explanatory statement ATSI_V2 | STAGE 3 Easy to read Explanatory statement ATSI_V2.docx | 31/03/2022 | V2 | 116.9 KB |
| Explanatory Statement | STAGE 3 Long EXPLANATORY STATEMENT ATSI_V2 | STAGE 3 Long EXPLANATORY STATEMENT ATSI_V2.docx | 31/03/2022 | V2 | 118.9 KB |
| Explanatory Statement | STAGE 3 FINAL Easy to read Explanatory statement deaf_V2docx | STAGE 3 FINAL Easy to read Explanatory statement deaf_V2docx.docx | 31/03/2022 | V2 | 117.5 KB |
| Explanatory Statement | STAGE 3 Easy to read Explanatory statement disability_V2 | STAGE 3 Easy to read Explanatory statement disability_V2.docx | 31/03/2022 | V2 | 117.3 KB |
| Explanatory Statement | STAGE 3 Long EXPLANATORY STATEMENT disability_V2 | STAGE 3 Long EXPLANATORY STATEMENT disability_V2.docx | 31/03/2022 | V2 | 119.0 KB |
| Explanatory Statement | STAGE 3 Easy to read Explanatory statement sex workers_V2 | STAGE 3 Easy to read Explanatory statement sex workers_V2.docx | 31/03/2022 | V2 | 116.8 KB |
| Explanatory Statement | Stage 3 Long EXPLANATORY STATEMENT St Kilda Gatehouse_V2 | Stage 3 Long EXPLANATORY STATEMENT St Kilda Gatehouse_V2.docx | 31/03/2022 | V2 | 118.6 KB |
| Explanatory Statement | STAGE 3 Easy to read Explanatory statement Refugee_aslyum seekers_V2docx | STAGE 3 Easy to read Explanatory statement Refugee_aslyum seekers_V2docx.docx | 31/03/2022 | V2 | 118.5 KB |
| Explanatory Statement | STAGE 3 Long EXPLANATORY STATEMENT Refugees_V2 | STAGE 3 Long EXPLANATORY STATEMENT Refugees_V2.docx | 31/03/2022 | V2 | 118.7 KB |
| Explanatory Statement | STAGE 3 Easy to read Explanatory statement Community group_V2 | STAGE 3 Easy to read Explanatory statement Community group_V2.docx | 31/03/2022 | V2 | 116.5 KB |
| Explanatory Statement | STAGE 3 Long EXPLANATORY STATEMENT Community group_V2 | STAGE 3 Long EXPLANATORY STATEMENT Community group_V2.docx | 31/03/2022 | V2 | 118.3 KB |

## Limited Disclosure or Deception

**B14 Will all participants in this research be fully informed about the true nature of the research?**

◉ Yes
○ No

## Consent Process

**B15 Please clarify how you will obtain informed consent from participants?**

*Please refer to the Monash Consent Form template and modify to suit your project.*

- ☐ Implied consent
- ☐ Verbal consent
- ☐ Opt-Out consent
- ☑ Consent form
- ☐ Waiver of consent
- ☐ Other

*Please explain the process by which the participants will give consent and how will they return the consent form to the researchers.*

Participants will be given access to a link for Qualtrics. This link will include the 'easy English' explanatory statement (with a link to the longer version imbedded), for the research being conducted and option to click yes to participate or no to not participate. Only those who click yes to participate will move on to complete the survey. This question is also considered a 'forced response' and participants must answer before being able to move further into the survey.
Please note the attached consent form is an example of how this first question in the survey looks. Please note that a separate survey will be created for each different vulnerable group and community group so that the explanatory statements correctly correspond, as do the videos in the survey.

*Please attach a copy of the Consent Form(s). If you are using multiple forms, please ensure you clearly label each document. To upload more than one document, click the "Upload Document".*

Documents

| Type | Document Name | File Name | Version Date | Version | Size |
|---|---|---|---|---|---|
| Consent Form | Consent form Example Refugee explanatory statement | Consent form Example Refugee explanatory statement.docx | 24/02/2022 | V1 | 16.8 KB |

## Capacity to Consent

**B16 Are all the participants in this project able to consent for themselves?**

- ○ Yes
- ◉ No

*Please explain how you intend to obtain informed consent. How will adequate information be provided to those who will give consent on their behalf?*

Participants may not be able to consent for themselves due to their disability or language as a barrier. For those with a disability and who may need assistance completing the survey, consent will be provided on their behalf from a guardian or care giver via the consent form imbedded into the survey. For those with language as a barrier, the explanatory statement, consent forms and full survey will be translated appropriately, or a member of the research team at the relevant partner organisation will be available to assist them verbally.

## Data Collection

**C1 This research will include the following data collection methods:**

- [x] a) Questionnaires / Surveys
- [ ] b) Interviews
- [ ] c) Photography / videography
- [ ] d) Focus groups
- [ ] e) Observations
- [ ] f) Psychological inventories
- [ ] g) Responses to tasks or stimuli
- [ ] h) Collection of biospecimens
- [ ] i) Administration of radiation
- [ ] j) Administration of a substance / use of a device
- [ ] k) Other

## Data Collection Details

*C1a Please provide details about the questionnaires / surveys and how these will be returned, e.g. paper based or online surveys, fully identifiable, coded surveys (potentially identifiable) or anonymous (can never be identified).*

***For anonymous online surveys:*** *also confirm below that you will set up a separate form to collect the required contact details and re-assure participants in the explanatory statement (at B13) that these details are collected separately and will not be linked to their survey responses.*

The surveys will be completed online using Qualtrics. They are completely anonymous and participants will not be able to be identifiable. Participants will be redirected to a separate survey to provide their contact details for gift card collection and to receive information of results of this research or to be involved in future research.

There will be a separate survey for each vulnerable population group (including for each language required- Dari and Sinahelse like in the Refugee group). We are still working on editing the community representative videos, as well as some of the animation videos. I have provided a link to the 'final videos' so far below (no visible in the attached survey). As you will see there are 6 context areas. The videos of the animation and the community representative includes the exact same information that is found in these final GP videos. However it is a different person providing the information (in the case of the community representative), and a different visual (in the case of the animation).
The survey for each vulnerable population will be exactly the same, only that the community representative video will be different (all the questions are the same). Where as the questions are slightly different in the community group survey as well as videos imbedded. The Community group survey will include videos delivered by various political parties explaining specific COVID-19 health messaging in lieu of a community representative (like seen in the vulnerable surveys). Thus, as we are aiming to assess trust and behavioural change after watching a video, we have included political persuasion questions in the community group survey. This is to help identify if it matters to the participant which political background the politician is from.
Please note that participants will be randomly allocated to complete a 'block of questions'. This means that they will only watch a total of 6 videos only (they will not be required to watch all videos across all blocks).
Attached you will find: Stage 3 vulnerable group survey (Aged Care Worker); Stage 3 contact details survey for vulnerable group- Aged Care Workers; Stage 3 community group survey; Stage 3 gift card draw survey for community group.

Link to videos on google drive: https://drive.google.com/drive/folders/1BX6ROpLPDouxurver3HAae2NKHrgnZWP?usp=sharing
**please contact megan.jepson@monash.edu if access permissions are still required.

*Please upload a combined copy of the questions (in one PDF) as the participants will see them. Ensure that any questions regarding gender align with Monash's guidelines on capturing gender data inclusively.*

Documents

| Type | Document Name | File Name | Version Date | Version | Size |
|---|---|---|---|---|---|
| Questionnaires / Surveys | Stage_3_Contact_details_survey_for_a_vulnerable_group-_Aged_Care_Workers | Stage_3_Contact_details_survey_for_a_vulnerable_group-_Aged_Care_Workers.docx | 24/02/2022 | V1 | 22.0 KB |
| Questionnaires / Surveys | Stage_3_Gift_Card_Draw_Survey_for_Community_Group | Stage_3_Gift_Card_Draw_Survey_for_Community_Group.docx | 24/02/2022 | V1 | 21.6 KB |
| Questionnaires / Surveys | Stage_3__Community_Group_Survey V2 FINAL 19052022(1) | Stage_3__Community_Group_Survey V2 FINAL 19052022(1).docx | 19/05/2022 | V2 | 66.0 KB |
| Questionnaires / Surveys | Stage_3_Vulnerable_Group_Survey-Aged_Care_Workers FINAL V2 19052022 (1) | Stage_3_Vulnerable_Group_Survey-Aged_Care_Workers FINAL V2 19052022 (1).docx | 19/05/2022 | V2 | 63.6 KB |

## Participant Groups and Data Collection Methods

**C4 Please list in the table below which of the method(s) indicated in C1 will be used for each group of participants listed at question B1.**

| Participant group (as listed in B1) | Relevant data collection method(s) |
|---|---|
| People living with disability & their carers | Qualtrics Survey completed either independently, with some assistance from a care giver or research team member online. |

----------------------------------------------------------------------------------------------------

*To add another group of participants, click the "Add Another" button.*

**C4 Please list in the table below which of the method(s) indicated in C1 will be used for each group of participants listed at question B1.**

| Participant group (as listed in B1) | Relevant data collection method(s) |
|---|---|
| Aged Care workers | Qualtrics Survey completed either independently, with some assistance from a care giver or research team member online. |

----------------------------------------------------------------------------------------------------

*To add another group of participants, click the "Add Another" button.*

**C4 Please list in the table below which of the method(s) indicated in C1 will be used for each group of participants listed at question B1.**

| Participant group (as listed in B1) | Relevant data collection method(s) |
|---|---|
| Refugee and asylum seekers | Qualtrics Survey completed either independently, with some assistance from a care giver or research team member online. The surveys will be available in language (Dari or Sinhalese) with a bicultural worker available to assist who is fluent in both English and the preferred language. |

----------------------------------------------------------------------------------------------------

*To add another group of participants, click the "Add Another" button.*

**C4 Please list in the table below which of the method(s) indicated in C1 will be used for each group of participants listed at question B1.**

| Participant group (as listed in B1) | Relevant data collection method(s) |
| --- | --- |
| Deaf, hard of hearing people | Qualtrics Survey translated into AUSLAN (with assistance from Expression Australia) will be completed independently or with assistance from a care giver or research team member online. |

-----------------------------------------------------------------------------------------------------

*To add another group of participants, click the "Add Another" button.*

**C4 Please list in the table below which of the method(s) indicated in C1 will be used for each group of participants listed at question B1.**

| Participant group (as listed in B1) | Relevant data collection method(s) |
| --- | --- |
| Aboriginal and Torres Strait Islander people | Qualtrics Survey completed either independently, with some assistance from a care giver or research team member online. |

-----------------------------------------------------------------------------------------------------

*To add another group of participants, click the "Add Another" button.*

**C4 Please list in the table below which of the method(s) indicated in C1 will be used for each group of participants listed at question B1.**

| Participant group (as listed in B1) | Relevant data collection method(s) |
| --- | --- |
| Street-based sex workers | Qualtrics Survey completed either independently, with some assistance from a care giver or research team member online. |

-----------------------------------------------------------------------------------------------------

*To add another group of participants, click the "Add Another" button.*

**C4 Please list in the table below which of the method(s) indicated in C1 will be used for each group of participants listed at question B1.**

| Participant group (as listed in B1) | Relevant data collection method(s) |
| --- | --- |
| General community | Qualtrics Survey completed independently online. |

-----------------------------------------------------------------------------------------------------

*To add another group of participants, click the "Add Another" button.*

## Research Procedures

**C5 Please provide details about what you are asking participants to do or what is to be done to them.**

*Include a step-by-step description of what participants will experience if they choose to take part in this project.*

Once recruited, participants will be given a link to the explanatory statement where they will then consent to participate. For those consenting to participate, they will then move though the online survey through Qualtrics asking them questions. All answers will be anonymous. At the completion of the survey, participants will be linked to a separate survey to opt in to be contacted for future research and to provide contact details to receive their gift card (where applicable).

**C6 How much time are you asking of participants and when will the time be required?**

*E.g. 30 min after class. Please include this information for each group of participants listed in B1 or for each different data collection method that will be used.*

The survey is estimated to take approximately 30-60mins, however does have the potential to take longer depending on the literacy level of the participate and ability to understand English.

**C7 Where will the data be collected and by whom?**

*E.g. Public library by the student researcher, University meeting room by the Chief Investigator.*

Data will be collected online via Qualtrics. iPads will be provided where necessary to supporting organisations to assist with completion of the survey. Paper-based copies of the survey for completion will be provided to sub-groups if specifically requested. Partner organisations include: Able Australia, Wallara, Yooralla, Donwood Community Aged Care Services, enliven, Expression Australia, Peninsula Health, and St Kilda Gatehouse. Where assistance to fill out the surveys is required, CI's and partner organisation contact people will be available to assist with data collection.

## Procedures and Qualifications

**C8 Does the research involve the administration of any tests or procedures that require particular qualifications?** ***(including surveys or psychological inventories)*****.**

- ○ Yes
- ◉ No

**C9 Does the research involve measures or procedures that are diagnostic or indicative of any medical or clinical condition, or any other situation of concern?**

- ○ Yes
- ◉ No

## Privacy and confidentiality

**H1 The research involves:**

- ☑ Identifiable personal information (e.g. consent forms with names etc..)
- ☐ Re-identifiable / CODED personal information
- ☐ Observation of participants
- ☐ None of the above

**H2 Will the personal information or observations be used without the consent or knowledge of the individuals?**

- ○ Yes
- ◉ No

## Data access and security

**I1 Describe the security arrangements for the storage of the data. Include details of where the data will be stored, how long it will be stored for and who will have access to the information?**

- *Ensure that the information noted here is consistent with the information included in the explanatory statement at question B13.*
- *In general, the minimum period for retention of research data is **5 years** from the date of publication. More information is available at this link.*

The collected responses will be stored in accordance with Australian Privacy Regulations, and will be stored on a secure cloud based server (Google Drive) for 5 years. After this it will be deleted. Only members of the research team will have access to collected responses.

**I2 Will a non-Monash third party have access to the data during this research?**

*E.g. using online survey tools such as Survey Monkey, translators or external data transcription or analysis/processing.*

- (•) Yes
- ( ) No

*Please explain how the participants are informed about this and how you will ensure that their privacy is protected during the data transfer process to the third party.*

Survey responses are collected through Qualtrics. Participants will be given the link to Qualtrics privacy policy for further information on how their information will stay protected.

**I3 Will the RAW DATA be made accessible in a data repository, registry or open source platform?**

- *Raw data refers to primary or unprocessed data. It does not include data that has been published.*
- *If Yes, please ensure that this is noted in the explanatory statement uploaded at question B13.*

- ( ) Yes
- (•) No

*Please explain why data will not be shared.*

Any data from this research will be shared publicly only in peer reviewed journals, conferences and reports back to the participants and partner organisations if they wish.

**I4 Are the data access and security arrangements detailed in the Explanatory Statement and Consent Form?**

- (•) Yes
- ( ) No

**I5 How will the hard copy and digital data be disposed of if it is no longer required?**

*Please ensure that your response refers to both hard copy and electronic data collected for this research.*

Digital data will be deleted from Google Drive after 5 years. If hard-copy surveys are completed, these will be shredded after 5 years.

## Research outcomes

**J1 Please indicate the format(s) in which the research will be published and/or communicated to participants or organisations.**

- [ ] Thesis
- [ ] Book / Book chapter
- [ ] Dataset
- [x] Report to organisation
- [ ] Other
- [x] Journal article
- [x] Conference
- [x] Report to participants
- [ ] Report to community or group
- [ ] Not Applicable

**J2 Please describe how participants and organisations will be able to access the results.**

*Ensure that the information noted here is consistent with the information included in the explanatory statement at question B13.*

Participants will have the option of requesting a summary of results at the completion of the survey.

**J3 In what format will others be provided with the results?**

- [x] In totally deidentified summary form, in which no individual can be identified.
- [ ] In deidentified summary form, but in a manner which may allow some individuals to be identified.
- [ ] In identified form, or in a manner which may allow some participants to be identified.
- [ ] Other

## Documents

### K1 Please attach other relevant supporting documents e.g. permission letters, amendment summary etc.

Documents

| Type | Document Name | File Name | Version Date | Version | Size |
|---|---|---|---|---|---|
| Supporting Documentation | MRFF CSADO Grant RRCSA000014_Partner Letter_Able Australia | MRFF CSADO Grant RRCSA000014_Partner Letter_Able Australia.pdf | 30/09/2021 | V1 | 875.2 KB |
| Supporting Documentation | MRFF CSADO Grant RRCSA000014_Partner Letter_Donwood | MRFF CSADO Grant RRCSA000014_Partner Letter_Donwood.pdf | 30/09/2021 | V1 | 209.9 KB |
| Supporting Documentation | MRFF CSADO Grant RRCSA000014_Partner Letter_Peninsula Health | MRFF CSADO Grant RRCSA000014_Partner Letter_Peninsula Health.pdf | 30/09/2021 | V1 | 169.7 KB |
| Supporting Documentation | MRFF CSADO Grant RRCSA000014_Partner Letter_SEHCP | MRFF CSADO Grant RRCSA000014_Partner Letter_SEHCP.pdf | 30/09/2021 | V1 | 832.4 KB |
| Supporting Documentation | MRFF CSADO Grant RRCSA000014_Partner Letter_St Kilda Gatehouse | MRFF CSADO Grant RRCSA000014_Partner Letter_St Kilda Gatehouse.PDF | 30/09/2021 | V1 | 159.0 KB |
| Supporting Documentation | MRFF CSADO Grant RRCSA000014_Partner Letter_Victorian Deaf Society | MRFF CSADO Grant RRCSA000014_Partner Letter_Victorian Deaf Society.pdf | 30/09/2021 | V1 | 431.0 KB |
| Supporting Documentation | MRFF CSADO Grant RRCSA000014_Partner Letter_Wallara | MRFF CSADO Grant RRCSA000014_Partner Letter_Wallara.pdf | 30/09/2021 | V1 | 225.9 KB |
| Supporting Documentation | MRFF CSADO Grant RRCSA000014_Partner Letter_Yooralla | MRFF CSADO Grant RRCSA000014_Partner Letter_Yooralla.pdf | 30/09/2021 | V1 | 162.7 KB |
| Supporting Documentation | Amendment Summary 19052022docx | Amendment Summary 19052022docx.docx | 19/05/2022 | V1 | 14.1 KB |

### K2 For projects from the School of Public Health

*Please upload a copy of the protocol with this application. Please note that any amendments to this protocol should include the new version numbers and should be submitted using track changes.*

## Declarations

**Signatures are not required. By submitting this form, you make the following declaration. You may delegate authority to submit applications, amendments and reports to other investigators listed on this project by 'sharing' the project with them. As CI, you will retain ultimate responsibility for the project including the content of all submissions.**

☑ By submitting this project, I/We, declare that I/We:

1. Accept responsibility for the ethical conduct of the research detailed above in accordance with the principles outlined in the National Statement and the Australian Code for the Responsible Conduct of Research.
2. Undertake to conduct this research project in accordance with the protocols and procedures outlined in this proposal as approved by MUHREC.
3. Will inform MUHREC of any proposed changes to the approved protocol by submitting an amendment prior to implementing the changes.
4. Will submit an annual report on the anniversary of approval and a final report when the research is completed.
5. Have read and agree to comply with the Monash Research Data Management Policy and have a plan for managing and/or sharing Research Data securely.
6. Understand and agree that study files and documents and research records and data may be subject to inspection by MUHREC, research governance officer, the sponsor or an independent body for audit and monitoring purposes.
